# HUPAN: a pan-genome analysis pipeline for human genomes

**DOI:** 10.1186/s13059-019-1751-y

**Published:** 2019-07-31

**Authors:** Zhongqu Duan, Yuyang Qiao, Jinyuan Lu, Huimin Lu, Wenmin Zhang, Fazhe Yan, Chen Sun, Zhiqiang Hu, Zhen Zhang, Guichao Li, Hongzhuan Chen, Zhen Xiang, Zhenggang Zhu, Hongyu Zhao, Yingyan Yu, Chaochun Wei

**Affiliations:** 1 School of Life Sciences and Biotechnology, Shanghai Jiao Tong University, 800 Dongchuan Road, Shanghai, 200240 China; 2 SJTU-Yale Joint Center for Biostatistics and Data Science, Shanghai Jiao Tong University, 800 Dongchuan Road, Shanghai, 200240 China; 3 Department of Radiation Oncology and Department of Oncology, Shanghai Medical College, Fudan University Shanghai Cancer Center, 270 Dong An Road, Shanghai, 200032 China; 4 Department of Pharmacology, Shanghai Key Laboratory For Translational Medicine, Shanghai Jiao Tong University School of Medicine, 227 South Chongqing Road, Shanghai, 200025 China; 5 Department of Surgery, Ruijin Hospital, Shanghai Key Laboratory for Gastric Neoplasms, Shanghai Jiao Tong University School of Medicine, 197 Ruijin Road, Shanghai, 200025 China; 6 Department of Biostatistics, Yale University, 60 College Street, New Haven, CT 06520 USA; 7 Shanghai Center for Bioinformation Technology, 1278 Keyuan Road, Pudong District, Shanghai, 201203 China

**Keywords:** Pan-genome, Core genome, Presence-absence variation (PAV), Genome assembly, Population-specific variation

## Abstract

The human reference genome is still incomplete, especially for those population-specific or individual-specific regions, which may have important functions. Here, we developed a HUman Pan-genome ANalysis (HUPAN) system to build the human pan-genome. We applied it to 185 deep sequencing and 90 assembled Han Chinese genomes and detected 29.5 Mb novel genomic sequences and at least 188 novel protein-coding genes missing in the human reference genome (GRCh38). It can be an important resource for the human genome-related biomedical studies, such as cancer genome analysis. HUPAN is freely available at http://cgm.sjtu.edu.cn/hupan/ and https://github.com/SJTU-CGM/HUPAN.

**Electronic supplementary material:**

The online version of this article (10.1186/s13059-019-1751-y) contains supplementary material, which is available to authorized users.

## Background

Single nucleotide variations (SNVs), small insertions and deletions (INDELs), and structural variations (SVs) of the human genome are routinely explored to study the genomic variations in biomedical studies. However, most of these studies are based on the human reference genome, which was built from several individuals, and only a consensus of these genomes was included [1]. Therefore, reference-based methods may miss some sequence variations within or between populations [2, 3]. Actually, previous studies have discovered various types of novel sequences, which are not present in the human reference genome [4–8]. For example, more than 3700 non-repetitive non-reference (NRNR) sequences were called from whole-genome sequence data of 15,219 Icelanders by de novo assembly of the unmapped reads into contigs [4]. In another study, by analyzing the unmapped reads from ~ 10,000 deep sequencing human genomes, Telenti et al. found that each genome carried an average of 0.7 Mb sequences that were not found in the human reference genome [6]. The Simons Genome Diversity Project reported high-quality genomes of 300 individuals from 142 diverse populations and suggested at least 5.8 Mb sequences from these genomes were not present in the human reference genome [9]. These novel sequences may harbor functional genomic elements that are ethnic specific, and may affect gene regulations or transcriptional diversity [2]. For example, a 766-bp non-repetitive non-reference sequence was found to have an association with myocardial infarction in Icelanders [4]. Adding these novel sequences into the human reference genome could improve the efficiency of mapping and variant calling process [9].

Over the past decade, due to the rapid decrease of sequencing cost, pan-genome analysis has become popular in bacteria [10, 11] and plants [12–16]. The approach of pan-genome analysis was first introduced by Tettelin et al. [17] in *Streptococcus agalactiae* study and aimed to reveal gene or gene family presence-absence variation (PAVs) within a species or a population. The pan-genome is composed of a “core genome” containing genes present in all individual genomes and a “distributed genome” (or dispensable genome, which is somewhat misleading as discussed by Marroni et al. [18]) containing genes in a subset of individuals of this species.

The first human pan-genome study was carried out in 2010, and only two representative genomes from Africa and Asia were analyzed [3]. In this study, about 5 Mb novel sequences absent in the reference genome (hg19 assembly) were detected for each individual and the total sequences absent in the reference genome were estimated to be 19~40 Mb, which might have been underestimated considering the study of 10 Danish trios [19]. In a subsequent study [2], re-analysis of the 5 Mb novel sequences from a Chinese individual showed that 3.7 Mb sequences could be aligned to GRCh38 human reference genome. In another Chinese genome HX1, 12.8 Mb sequences were detected not present in GRCh38 but 68% of these novel sequences could be found in Asian populations [2]. In a latest paper, Sherman et al. reported an African pan-genome [20]. It contained about 300 Mb unique sequences missing in the human reference genome. Notably, most of these novel sequences were individual-specific, and only 81 Mb sequences were shown in two or more individuals. These studies indicated the significance of population-specific genome diversity. The possibility of these non-reference genomic regions to be the driver mutations for some diseases, especially for those dominated by a certain specific ethnic group, is worth our effort to investigate.

The explosive growth of human whole-genome sequencing data brings significant challenges and tremendous opportunities to study the pan-genome of a specific population [21]. However, constructing the pan-genome sequences from hundreds of individual genomes is a huge challenge. Recently, we reported a tool EUPAN [22] based on a “map-to-pan” strategy and applied it to more than 3000 rice genomes [13]. Nevertheless, due to the large size of the human genome, EUPAN cannot be applied for human pan-genome analysis because of the huge memory size requirement of the de novo assembly step (more than 500 Gb memory is needed to assemble a human genome from a 30-fold sequencing data. See more details in Additional file 1: Supplementary methods). Several previous studies reported non-reference genome sequences using the approach of pseudo de novo assembly [4, 6, 8, 20]. Instead of using all reads, only the unmapped reads were extracted to conduct de novo assembly [8, 20]. We compared the assembled results using all reads and unmapped reads with simulated sequencing data, and suggested that pseudo de novo assembly method may underestimate the size of non-reference sequences and produce more misassembled sequences at the meantime (Additional file 1: Table S1). If all reads were used, aligning hundreds of assembled genomes to the human reference genome to extract the non-reference sequences and distinguishing the non-human sequences contaminated in sampling, sequencing, and other procedures are other challenges that need to be addressed.

In this paper, we present a HUman Pan-genome ANalysis (HUPAN) tool and apply it to analyze 275 Han Chinese genomes, including 185 newly sequenced and 90 assembled genomes [23]. HUPAN can also be applied to other eukaryotes with big genome sizes similar to human.

## Results

**Fig. 1:**
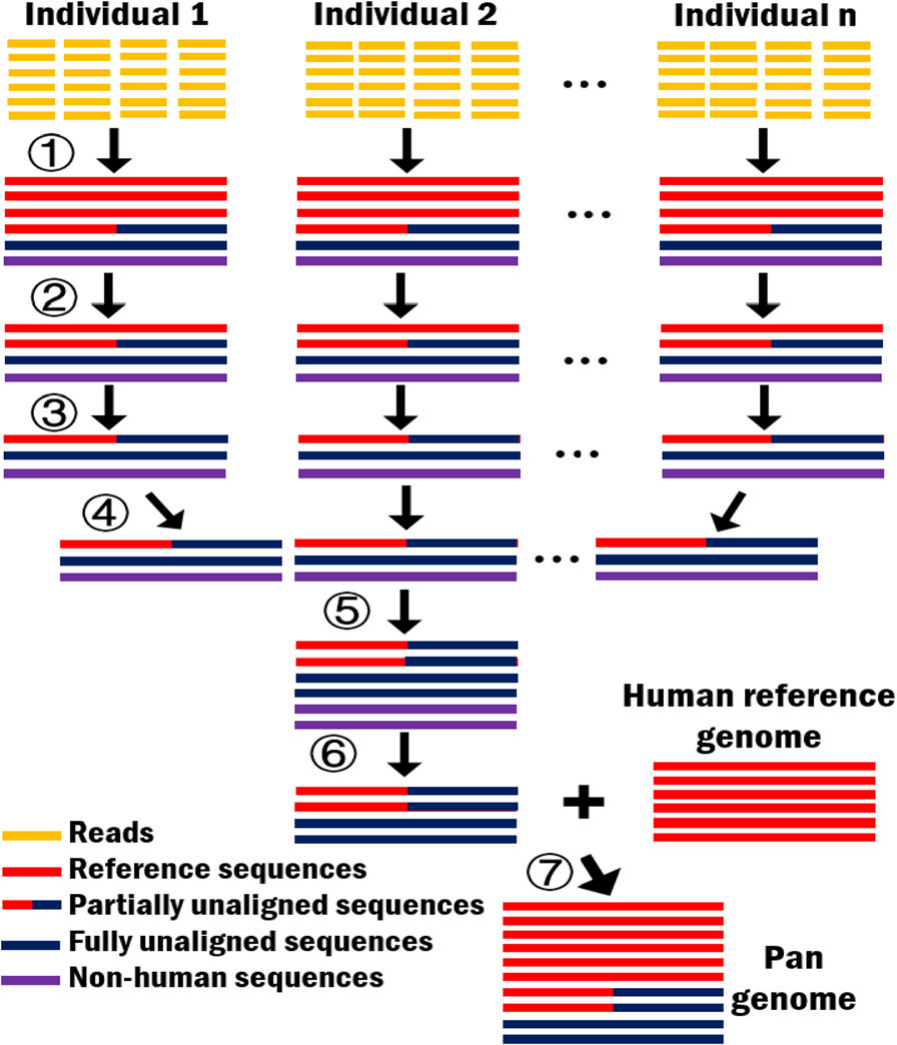
System diagram of pan-genome construction subsystem in HUPAN. Seven processes include as follows: ① de novo assembly all reads into contigs, ② removing contigs similar to the human reference genome, ③ extracting unaligned sequences (including fully unaligned sequences and partially unaligned sequences), ④ merging unaligned sequences from multiple individuals, ⑤ removing redundant sequences, ⑥ removing potential contaminations, and ⑦ constructing pan-genome combining the human reference genome and novel sequences

We have developed a pan-genome analysis system, HUPAN, for analyzing deep sequencing data of a large number of human individuals. Similar to EUPAN, HUPAN utilizes the “map-to-pan” strategy to determine gene PAVs for each individual. It has a number of distinct improvements listed as follows: (1) de novo assembly of each individual genome is performed with SGA [24], a low memory requirement program; (2) a faster non-reference sequences extracting strategy is created; (3) both fully unaligned sequences and partially unaligned sequences are considered to generate the non-reference genomic regions; and (4) a rigorous screening process is proposed to distinguish non-human sequences from non-reference sequences. Figure 1 shows the system diagram of pan-genome construction subsystem in HUPAN. We will decipher results of each step in details in the following based on 185 deep sequencing as well as the 90 assembled Han Chinese genomes.

### De novo assembly of 185 deep sequencing genomes

We selected SGA instead of SOAPdenovo2 [25] due to its high assembly quality and distinctly low memory consumption. We first optimized the assemble parameters based on simulation data (Additional file 1: Supplementary methods and Table S2). Then, we conducted de novo assemble for the 185 newly sequenced Han Chinese genomes using all reads (see the “Methods” section). As a result, the average size of the assembled 185 genomes was 2,720,566,559 ± 7,126,135 bp and the average size of contigs N50 was 8042 ± 387 bp (Additional file 1: Figure S1).

### Extracting non-reference sequences from assembled contigs

**Fig. 2:**
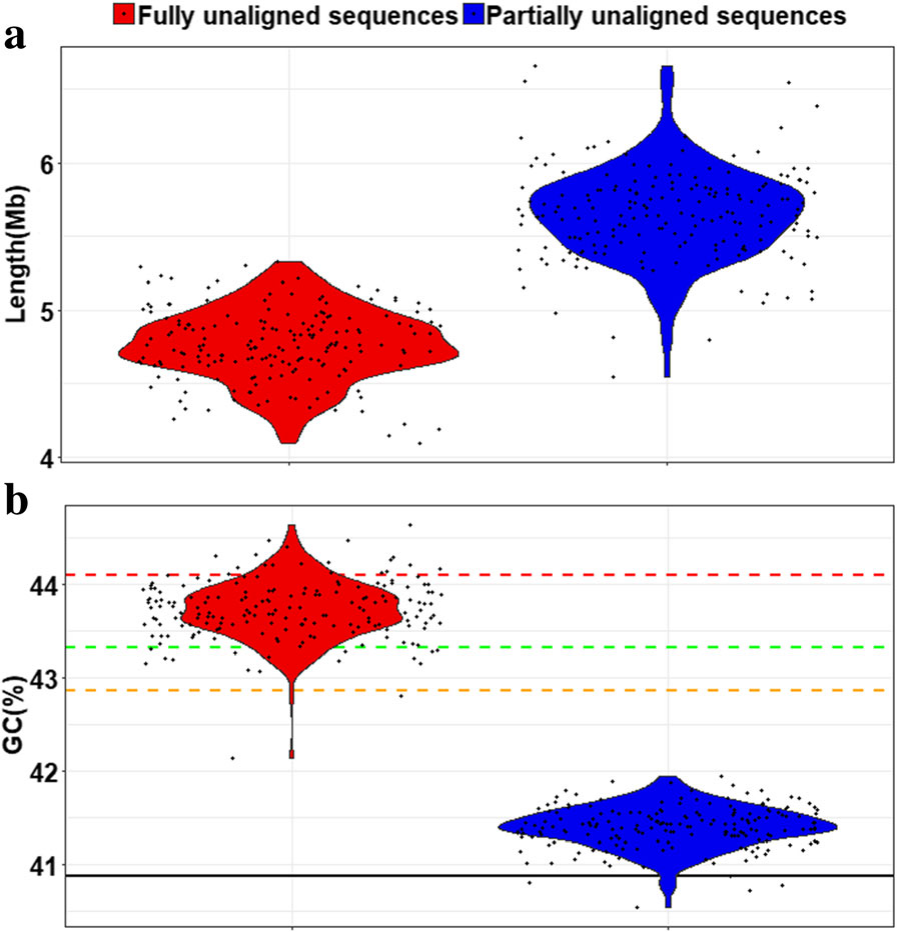
Summary of non-reference sequences for individual genomes. **a** The total length (Mb) and **b** the GC content (%) of unaligned contigs (including fully unaligned sequences and partially unaligned sequences) obtained for each individual after removing potential contamination. In **b**, the solid black line represents GC content of the primary sequence in GRCh38 (40.87%); the dotted lines represent GC content of novel sequences of YH genome [26] (red, 44.11%); 5.8 Mb novel contigs from SGDP [9] (green, 43.43%) and novel sequences of NA18507 genome (orange, 42.87%). The width of each plot indicates the frequency of samples with a given length or GC content

In HUPAN, we proposed a hierarchical strategy to extract the non-reference sequences (see the “Methods” section). Comparing with EUPAN, this new strategy could severely reduce both CPU time and memory consumption but with little loss in precision (Table 1 and Additional file 1: Figure S2). After discarding the potential contamination, ~ 5 Mb fully unaligned sequences and ~ 6 Mb partially unaligned sequences for each individual were obtained (Fig. 2a). Obvious stratification was observed in the fully unaligned sequences before removing contamination sequences (Additional file 1: Figure S3), which were mainly from the bacterium *Helicobacter pylori*, one majority infectious agent associated with gastric diseases in several individuals (Additional file 1: Figure S4). In addition, the GC content (%) of fully unaligned sequences was slightly higher than that of partially unaligned sequences. This result is consistent with previous studies [3, 9, 26] (Fig. 2b).

**Table 1:** Comparing of HUPAN and EUPAN in the procedure of extracting non-reference sequences of an individual genome

	HUPAN	EUPAN
# raw contigs (> 500 bp)	610,537	610,537
raw contigs length (bp)	2,709,735,693	2,709,735,693
# contigs after filtering	24,150	–
contigs length after filtering (bp)	76,168,613	–
# misassemblies	1037	1050
Misassembled contigs length (bp)	5,483,408	5,657,999
# Fully unaligned contigs	5371	5394
Fully unaligned contigs length (bp)	5,000,779	5,014,971
# Partially unaligned contigs	1187	1197
Partially unaligned contigs length (bp)	5,435,999	5,628,509
CPU time (hours)	42	275
Maximum memory (Gb)	92	250

After the non-reference sequences were merged, they were clustered to remove the redundant sequences across individuals. We obtained 52.90 Mb fully unaligned sequences and 46.76 Mb partially unaligned sequences. More than 20 Mb of the 52.90 Mb sequences were classified into microorganisms (Additional file 1: Figure S5a). Majority of the partially unaligned sequences were classified into human and other primates (Additional file 1: Figure S5b), indicating these sequences are indeed from human genomes. After removing sequence contaminations from microorganisms and non-primate eukaryotes, we identified 28,622 fully unaligned sequences, with a total length of 30.72 Mb and 8320 partially unaligned sequences, with a total length of 46.63 Mb (Additional file 1: Figure S6).

### Characterizations of fully unaligned sequences

**Fig. 3:**
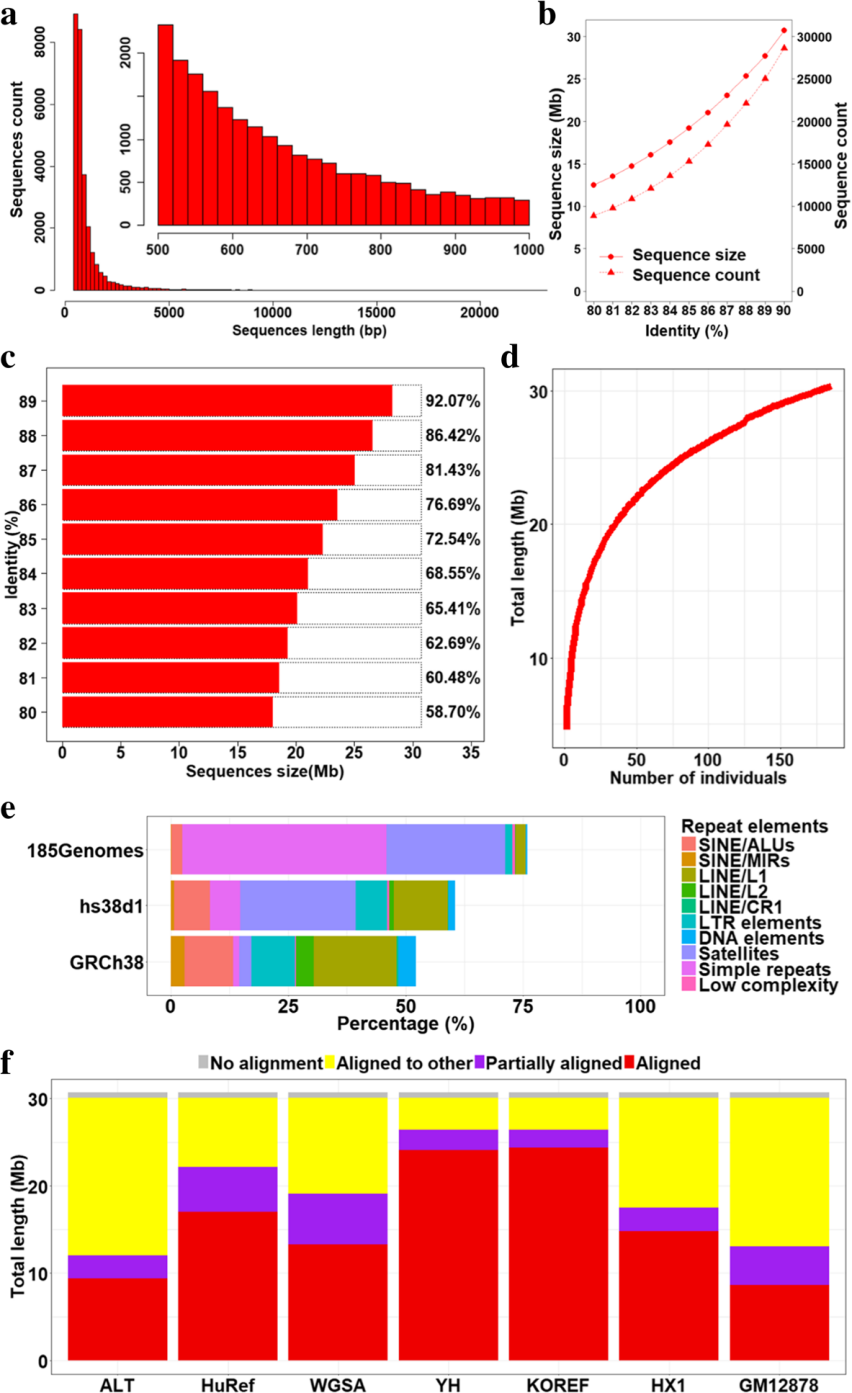
Characterization of sequences fully unaligned to GRCh38 primary assembly sequences in 185 deep sequencing Han Chinese genomes. **a** Length distribution of fully unaligned sequences. **b** The total length of fully unaligned sequences (Mb) obtained by using lower identity (80–90%) to remove redundant sequences. **c** The sequence count and sequence size when aligning the sequences to GRCh38 primary assembly sequences with lower sequence identity (80–90%). **d** Simulation of the total fully unaligned sequences using different numbers of individuals. **e** The percentage of repeat elements resulted from RepeatMasker, “hs38d1” is 5.8 Mb novel sequences from SGDP, and “GRCh38” is the primary assembly sequences of the human reference genome GRCh38. The RepeatMasker masked result of GRCh38 was downloaded from http://www.repeatmasker.org/species/hg.html. **f** Validation of fully unaligned sequences by aligning to other available human sequences (≥ 90% identity). “Aligned” defines the sequences that could be aligned to the target sequences, “Partially aligned” defines the sequences that could be partially aligned to the target sequences, “Aligned to other” defines the sequences that could not be aligned to the target sequences but could be aligned to other six available human sequences, and “No alignment” defines the sequences that could not be aligned to anyone of the seven data sets

For the fully unaligned sequences, their length distribution is shown in Fig. 3a. Among them, 7553 (26.39%) sequences had lengths > 1 kb and 94 had lengths > 10 kb. The amount of fully unaligned sequences decreased as the sequence identity threshold went down, and there were only 8849 sequences (12.51 Mb in total) left when the identity threshold decreased to 80% (Fig. 3b). A portion of these sequences could be aligned to the GRCh38 primary assembly sequences with different identities (from 80% to 90%) (Fig. 3c), suggesting that they were moderately similar to the reference genome. We estimated the effect of the number of individuals on the total length of the fully unaligned sequences. As showed in Fig. 3d, the total length of fully unaligned sequences from 50 individuals was ~ 22 Mb, and adding another 50 individuals only increased less than 4 Mb sequences. When the number of individuals was increased to 150, the total length was ~ 29 Mb. This indicated further increasing the number of individuals slightly extended the total length of fully unaligned sequences. The percentage of simple repeats and satellites in these sequences were significantly higher than that of the GRCh38 primary assembly sequences while the percentage of SINEs (short interspersed nuclear elements, including ALU and MIR) and LINEs (long interspersed nuclear elements, including L1, L2, and CR1) were lower than that of the GRCh38 primary assembly sequences (Fig. 3e). In addition, 6547 (22.90%) of 28,588 non-reference sequences were present in all 185 individuals (Additional file 1: Figure S7).

These sequences were validated by the additional sequences of GRCh38 reference genome and previously published human genome assemblies [2, 26–30]. Most of the sequences (30.07 Mb) could be fully or partially aligned to the above genomes at 90% identity (Fig. 3f and Table 2). In particular, 24.10 Mb (78.46%) could be aligned to the YH genome [26], which is the first assembled Asian individual, and 24.37 Mb (79.35%) could be aligned to the KOREF genome [29], which is from a South Korean individual. To our surprise, the percentage (48.0%) of sequences that could be aligned to HX1 genome [2], which is from a Chinese individual, is lower than that of the HuRef genome (55.3%) [28]. There were only 8.64 Mb (28.11%) that could be aligned to the GM12878 [30], which was originated from European, indicating a significant portion of these 30.72 Mb sequences may be Chinese-specific or East Asian-specific. Overall, there were only 646.23 Kb that could not be aligned to all the above genomes at all, and this indicates that the vast majority of the fully unaligned sequences were valid human DNA sequences.

**Table 2:** Validation of fully unaligned sequences by aligning to other existing human sequences (>= 90% identity). The last line showed the length of sequences unaligned to any of existing genomes

Assembled genomes	Alignment (bp)	Partially unaligned (bp)	Fully unaligned (bp)
ALT	9,383,032	2,641,297	18,693,461
HuRef	17,031,675	5,138,981	8,547,134
WGSA	13,261,237	5,836,552	11,620,001
YH	24,099,632	2,330,394	4,287,764
KOREF	24,374,934	2,012,184	4,330,672
HX1	14,797,305	2,748,577	13,171,908
GM12878	8,635,247	4,473,988	17,608,555
No alignment	646,233		

### Characterizations and validations of novel predicted genes

In total, 167 full-length novel genes were predicted on non-redundant non-reference sequences from 185 deep sequencing individuals (Additional file 1: Figure S8). The median length of novel predicted genes (614 bp) was shorter than that of those genes located in the human reference genome (27.04 Kb). We validated the novel predicted genes by two RNA-Seq data sets. At the threshold of 95% coverage, 46.71% of the full-length novel genes (78/167) were expressed in one or more of the 90 gastric tissues. When the threshold of coverage decreased to 80%, 120 novel genes were validated (Additional file 1: Figure S9). In addition, about 30% (50/167) were expressed in at least one of the 1001 publicly available RNA sequencing datasets.

### PAV analysis of 185 deep sequencing genomes

**Fig. 4:**
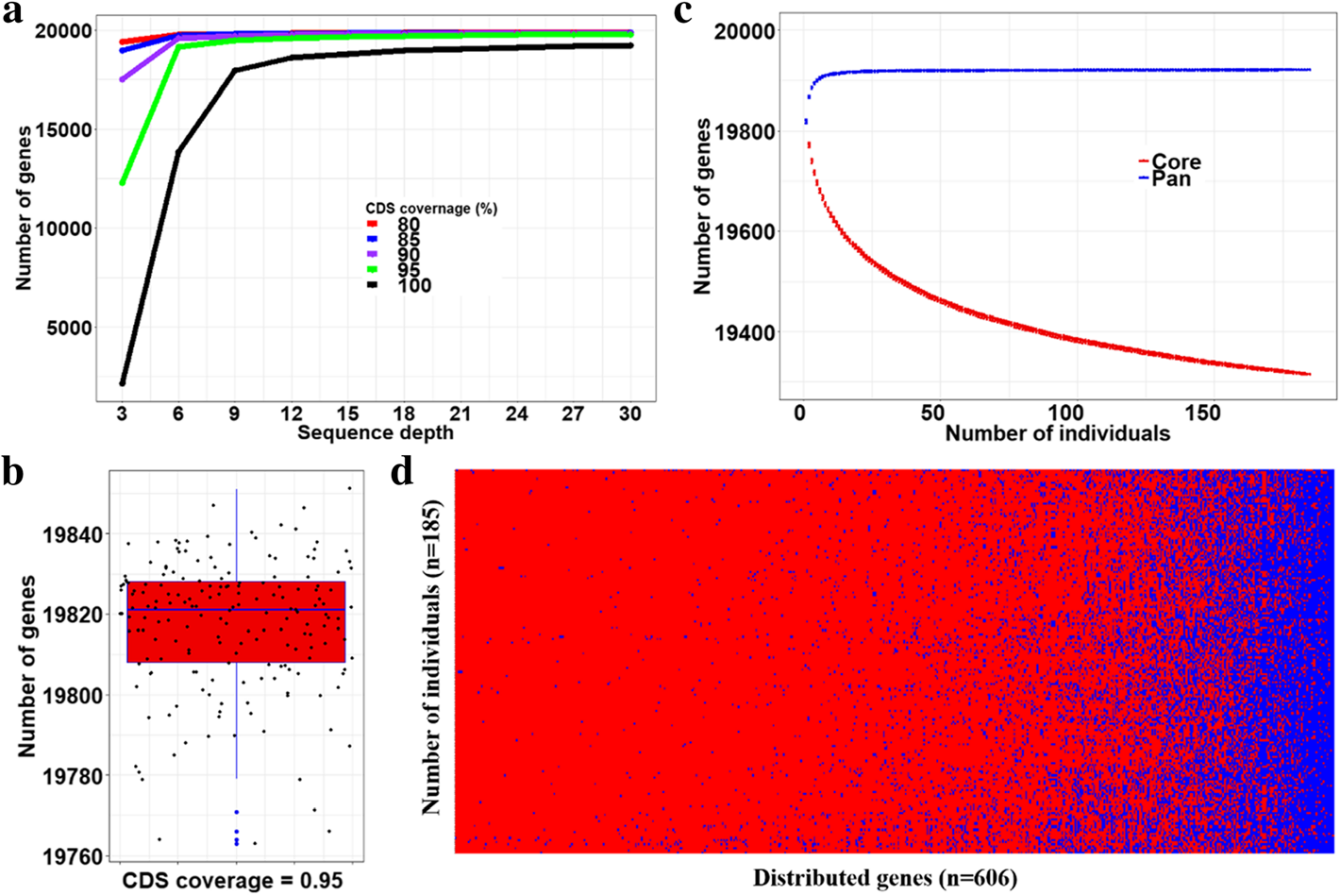
PAV profile analysis of 185 deep sequencing Han Chinese genomes. **a** The number of genes present in an individual using different CDS coverage threshold (80%, 85%, 90%, 95%, and 100%) versus the sequencing depth. **b** The gene PAV distributed across 185 individuals with the CDS coverage of 0.95. **c** The number of core genes and the total number of genes in the pan-genome determined with different number of individuals. Each time we randomly increased one individual and calculated the number of core genes and the total number of genes. These processes repeated 100 times. **d** PAV profile of 606 distributed genes. The red indicated gene presence and the blue indicated gene absence

In total, there were 19,921 protein-coding genes, including 19,754 genes located on human reference genome and 167 novel predicted genes. We used the reads from one individual GCH1N00001G to explore the relationship of reads’ depth and the CDS coverage (the percentage of coding sequence (CDS) of a gene was covered by at least one mapped reads for each individual genome) on individual gene PAV. The number of genes present in the individual was increased as the sequencing depth was increased, and the gene number tended to be stable when the depth was larger than six (Fig. 4a). The gene number was decreased by increasing the threshold values of CDS coverage. We selected CDS coverage of 95% to determine the core genes (the genes present in all individuals) and distributed genes (the genes absent in at least one individual), since no big change had been observed when CDS coverage was decreased to lower than 95%. On average, there were 19,817 (ranging from 19,763 to 19,851) genes in one individual genome (Fig. 4b), and the core genome included 19,315 (96.88%) genes (Fig. 4c). In total, there were 606 distributed genes (Fig. 4d), of which 490 (80.85%) were GRCh38 reference genes, and the rest 116 genes were the novel predicted genes. The percentage (69.46%) of distributed genes in 167 novel predicted genes were significantly higher than that of the reference genes (2.48%). Of the 490 distributed genes on the reference genome, several were known common gene deletion polymorphisms [31]. For example, ten genes showed common gene deletion polymorphisms with the coding exons missing; six of these genes (*UGT2B17*, *UGT2B28*, *LCE3C*, *GSTM1*, *OR51A2*, and *AR4F5*) were considered as distributed genes across 185 deep sequencing genomes.

### Pan-genome analysis of 90 Han Chinese genomes

We included a set of assembled genomes derived from 90 Han Chinese genomes [23]. By HUPAN program, in total, we obtained 318.66 Mb fully unaligned sequences (≥ 500 bp) and 151.84 Mb partially unaligned sequences from 90 individuals. On average, there were 3.54 Mb fully unaligned sequences and 1.69 Mb partially unaligned sequences for each individual. Few sequences of either fully unaligned sequences or partially unaligned sequences were classified into microorganisms and non-primate eukaryotes. Since the genomic DNA used for sequencing was extracted from the cell lines [23], there was few or no microbial contamination.

After removing redundant sequences and potential contaminations, there were 10.37 Mb fully unaligned sequences left (Additional file 1: Figure S10). When we aligned these sequences to the 30.72 Mb fully unaligned sequences from 185 deep sequencing genomes with a threshold of sequence identity ≥ 90%, 4.45 Mb (42.95%) and 7.21 Mb (69.57%) could be aligned when sequence length coverage was set to 100% and 80%, respectively (Additional file 1: Figure S11), indicating the high consistency of non-reference sequences between 185 deep sequencing genomes and 90 assembled Han Chinese genomes. Of the 79 novel predicted genes (Additional file 1: Figure S12), eight (10.13%) were also predicted in 185 deep sequencing genomes. When the gene sequence identity level was decreased to 80%, 30 (37.97%) of 79 novel genes were also predicted in 185 deep sequencing genomes.

### Comparison of African pan-genome and Han Chinese pan-genome

**Fig. 5:**
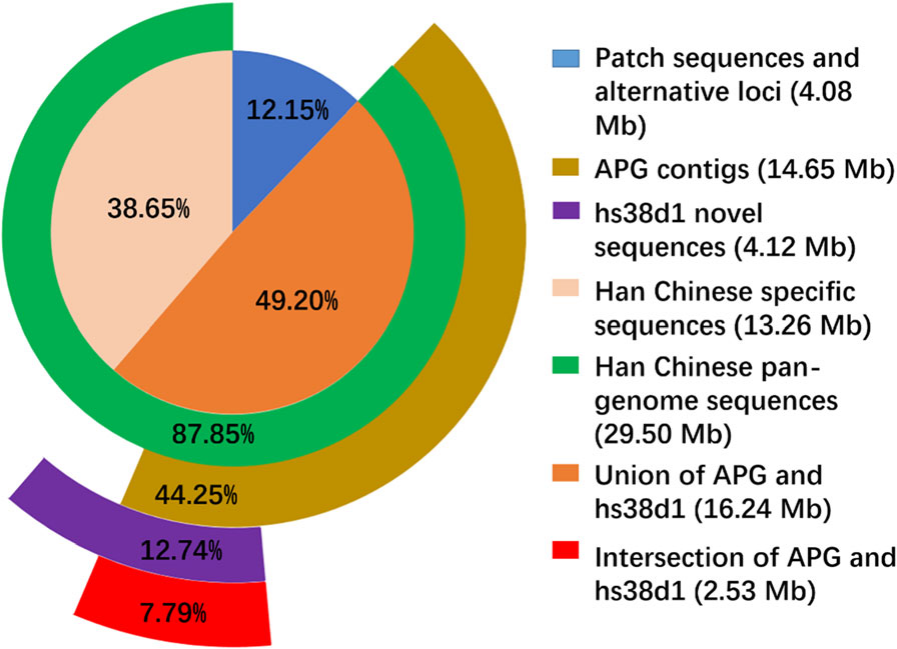
Comparison of 33.58 Mb novel sequences from 275 Han Chinese genomes with other human genome data sets. Other human genome datasets include the patch sequence and alternative loci of GRCh38, novel sequences of African pan-genome (APG), and the novel sequences of hs38d1

Firstly, we merged the novel sequences and novel predicted genes from 275 Han Chinese genomes, and obtained 33.58 Mb non-redundant sequences and 215 non-redundant novel genes. Of these sequences, 4.08 Mb (12.15%) could be aligned to patch sequences and alternative loci with an identity ≥ 90% that covered ≥ 80% of the sequence (Fig. 5). A total number of 27 novel genes could be aligned to patch sequences and alternative loci. In the rest 29.50 Mb novel sequences, 4.12 Mb was validated by hs38d1 decoy novel sequences and 14.65 Mb was intersected with African pan-genome contigs. In addition, 13.26 Mb novel sequences were specific to these 275 Han Chinese individuals, representing population-specific or individual-specific sequences (Fig. 5).

In order to compare our data sets with APG contigs, we aligned all 29.5 Mb novel sequences in the Han Chinese pan-genome to APG contigs in a reciprocal manner. We found nearly half (49.67%) of our 29.50 Mb novel sequences intersected with APG contig at 90% identity and at least 80% of the sequence length. Among these, about one third (9.9 Mb, 33.57%) of these novel sequences were exactly the same as APG contigs (Additional file 1: Figure S13). This indicates that these sequences were common in Han Chinese and African individuals but were missing in the reference genome. In addition, 25.21% of our data set was Han Chinese specific (< 10% of sequence length could overlap with APG contigs) (Additional file 1: Figure S13). This underscores the importance and necessity of constructing population-specific or individual-specific pan-genome. Therefore, more accurate national or population-specific pan-genome should be created for clinical and public health genetics [19].

## Discussion

The ongoing improvements of high-throughput sequencing technology and analytic capabilities promote the availability of DNA sequencing data. A number of re-sequencing projects have completed and resulted in high volumes of whole-genome sequencing data [4, 6, 9]. These datasets, especially those deep sequencing data from large cohorts, make it possible to carry out population-scale pan-genome analysis, such as the individuals within a certain geographical range or with a certain disease. Currently, hundreds of individual assembled human genomes are available at NCBI (ftp://ftp.ncbi.nlm.nih.gov/genomes/genbank/vertebrate_mammalian/Homo_sapiens/latest_assembly_versions/) and other databases [21, 23]. These data provide a great opportunity to understand more complex genetic diversity of human genomes and gain insight on population-specific variations, which are important for clinical or public health [19]. We have shown a strategy based on high-depth sequencing, de novo assembly, gene prediction of novel sequences, and maping raw reads to pan-genome to determine the gene PAV in a large number of human individuals. We demonstrated the power of our pipeline on 185 newly sequenced and 90 assembled Han Chinese genomes.

In this manuscript, we considered the non-reference genes. This approach also could be extended to the study of other genomic variations, such as copy number variations and other structural variations. For example, the misassembled contigs could be further analyzed to call these large structural variations, which were less accessible by reference-based variation calling tools. In addition, we used two independent cohorts to show the power of HUPAN for pan-genome analysis. All individuals were sampled from Han Chinese population, and this analysis could be extended to other populations to capture the global genetic variations and also various tumors to explore the dynamic variations of cancer genomes.

DNA contamination from other organisms may lead to imprecise outcome and should be considered in any sequencing project [32]. This is particularly important when we focus on the non-reference sequences. There are several possible sources of contaminants, such as biological source and DNA present in reagents or instruments [33]. In order to get high confidence non-reference sequences derived from human genome rather than contamination, we proposed a strict filtering step to drop potential contamination sequences as many as possible. We used a local alignment method to classify and exclude the sequences labeled as microorganisms or non-primate eukaryotes. The major source of non-human sequences was microorganisms, and majority of remaining sequences were labeled as human.

Recently, several novel genomic sequencing and assembly technologies have been developed to gain insight into the genomic dark matter in the human genome. For example, long-read sequencing technologies, such as single-molecule real-time (SMRT) sequencing from Pacific Biosciences (PacBio) or nanopore-based sequencing from Oxford Nanopore Technologies, provide a new opportunity to de novo assemble a high-quality genome. Several publications have reported assembled results of individual human genomes from these long-read length-sequencing platforms [2, 29, 30]. However, the significantly higher error rates and higher costs of long-read length sequencers prohibit applying it to population-scale sequencing. Many researchers move forward to the goals of reducing the cost, improving the accuracy and developing new algorithms for long-read length sequencers [34]. This will enable rapidly advancing the fields including constructing complete reference genomes, more comprehensive variant identification, and better understanding of human genomes. There are a tremendous number of new opportunities for further understanding of the human pan-genome with the progress of these long-read length-sequencing technologies.

## Conclusion

A pipeline (HUPAN) is proposed to build the human pan-genome sequences and to determine the gene PAV profile by mapping all reads to the constructed pan-genome sequences. We applied HUPAN to 185 deep sequencing genomes and 90 assembled genomes and detected about 33.58 Mb genome novel sequences, which encode at least 215 novel protein-coding genes, missing in the GRCh38 primary assembly sequences. Among these, 4.08 Mb sequences and 27 protein-coding genes can be aligned to patch sequences and alternative loci in GRCh38, respectively. Overall, there are 29.5 Mb novel sequences and at least 188 novel protein-coding genes in the Han Chinese pan-genome. This extends the comprehensive human genetic variation catalogs and highlights the importance of detecting non-reference sequences. HUPAN is a useful tool for capturing complexity of the human genome, and the constructed pan-genome can be an important resource for a wide range of human genome-related biomedical studies, such as cancer genome analysis.

## Methods

This section summarizes the components of our computational pipeline for human pan-genome analysis. More details are provided in the Additional file 1: Supplementary methods.

### De novo assembly

De novo assembly is one of the important tasks in pan-genome analysis, which provides the capacity of detecting sequences missing in the current reference genome. In EUPAN, SOAPDenovo2 was used to assemble individual genome. However, due to the large size of the human genome, assembling an individual genome from a 30-fold depth sequencing data requires more than 500 Gb of memory (Additional file 1: Table S4), which prohibits assembling hundreds of individual genomes in practice. After comparing several de novo assembly tools for next-generation sequencing data for large-sized genomes (Additional file 1: Supplementary methods), we selected SGA (String Graph Assembler) [24] due to its high assembly quality and low memory consumption. We obtained optimized parameters of SGA (Additional file 1: Table S2) on a simulation data and ran SGA with this parameter setting on 185 deep sequencing genomes in parallel.

### Identification of non-reference sequences

Building pan-genome sequences from individual assemblies is another challenging task. We adopted a strategy based on a well-assembled and well-annotated reference genome. In order to obtain non-reference sequences from individual genomes, contigs unable to be aligned to the GRCh38 primary assembly sequence (with identity cutoff of 90%) were collected for each individual. Due to the large size of the human genome, this process using QUAST [35] directly is time-consuming and requires a huge amount of memory (Table 1). In order to speed up this step, we developed a two-step strategy: discarding the contigs highly similar with the reference genome followed by extracting non-reference sequences (Additional file 1: Supplementary methods). In HUPAN pipeline, we focused on two types of non-reference sequences: fully unaligned sequences and partially unaligned sequences. Fully unaligned sequences are defined as contigs with no alignment to the reference sequence while partially unaligned sequences are defined as contigs with at least one alignment and at least one unaligned fragment longer than a defined threshold (default, 500 bp). After obtaining individual non-reference sequences, we merged them and removed redundant sequences by CDHIT [36] with the identity cutoff of 90%. We discarded those sequences whose best match were microorganisms including bacteria, fungi, archaea, and viruses and non-primate eukaryotes including all plants and non-primate animals, which could reflect possible contaminations (Additional file 1: Supplementary methods).

In order to understand the characteristics of the fully unaligned sequences, we ran CDHIT to further remove redundant sequences with lower identity levels and explored the similarity among the fully unaligned sequences. We decreased the threshold of sequence identity to explore the similarity between the fully unaligned sequences and the human reference genome. To estimate whether the fully unaligned sequences would continue to grow as the individuals increased, we added the fully unaligned sequences of each individual to run another round of clustering and remove the redundant sequences until the fully unaligned sequences from all individuals have merged into the non-redundant sequence dataset. We explored the repetitive elements of these sequences by RepeatMasker (http://www.repeatmasker.org/) and compared them with that of reference genome (both the primary assembly sequences and decoy sequences (hs38d1)) to characterize the compositions of repetitive sequences in fully unaligned sequences. Finally, we aligned these fully unaligned sequences to the patch sequence, alternative loci and decoy sequences (hs38d1) [9] as well as existing assembled individual genomes [2, 26–30] to determinate whether the fully unaligned sequences could be identified in other individuals.

### Construction and annotation of pan-genome sequences

We further removed redundancy between fully unaligned sequences and partially unaligned sequences and derived a non-redundant non-reference sequences dataset with a total size of 66.04 Mb (28,588 sequences). We added this dataset of non-reference sequences into GRCh38 primary assembly sequences to construct the pan-genome of 185 newly sequenced Han Chinese.

The annotation of GRCh38 primary assembly sequences and non-reference sequences were independent. The gene/transcript annotation of GRCh38 primary assembly sequences was based on GENCODE [37] (Release 26). In total, there are 19,817 protein-coding genes in the annotation database. If a gene has multiple transcripts, only the transcript with the longest open reading frame (ORF) was selected as a representative. Since all genes located in chromosome Y were absent in all female individuals, we excluded 63 genes in chromosome Y.

Protein-coding genes on non-reference sequences were predicted using MAKER [38] (Additional file 1: Supplementary methods). After stringent filtering processes to remove potential redundancy, 167 full-length genes were obtained (Additional file 1: Figure S8). These 167 novel genes predicted from the non-reference genome sequences were combined to the genes from the reference human genome, constructing 19,921 protein-coding genes for the human pan-genome based on the 185 deep sequencing Han Chinese genomes. These 19,921 genes were applied to generate the gene PAV profile of the 185 newly assembly Han Chinese genome.

### Determination of gene PAV profile

All reads of each individual were mapped to the pan-genome sequences using Bowtie2 [39, 40] with default parameters. SAMTools [40] and Picard software (http://broadinstitute.github.io/picard/) were used to sort and index the alignment files. The coding coverage and gene body coverage of each gene in each individual were calculated from the sorted “.bam” files. We used gene coverage and/or CDS coverage (covered bases in ORF / ORF length) to determine whether a gene was present in one individual. To confirm that the sequencing depth of 30-fold was sufficient to analyze the gene PAV of one individual, we selected the individual GCH1N00001G and sampled the alignment result to form subsets of 3- to 27-folds with a step size of 3. The subsets with different coverage were used to determine gene PAV analysis under different CDS coverages.

### Determination of core and distributed genes

The core genes refer to the genes present in all individuals, and the distributed genes refer to the genes absent in at least one individual. We used the threshold of CDS coverage of 95% to determine gene PAV for each individual. Then, we decided the core gene set and distributed gene set.

### Application to 90 assembled Han Chinese genomes

The HUPAN pipeline could be applied to existing assembled human genomes or other WGS project as well. Ninety unrelated individuals with Chinese ancestry were sequenced and assembled [23]. We downloaded all the assembled scaffolds and applied the HUPAN pipeline to extract non-reference sequences, discard the potential contaminations and redundancy, predict novel genes, and characterize them according to the steps described in the previous section (Additional file 1: Supplementary methods).

## Additional file

Additional file 1: This file contains supplementary methods (section 1), supplementary figures (section 2), and supplementary tables (section 3). (DOC 3790 kb)

## Data Availability

HUPAN is implemented in Perl, R, and C++ languages, and the source code is freely available under the MIT license at http://cgm.sjtu.edu.cn/hupan/ and https://github.com/SJTU-CGM/HUPAN [41]. An archival version of HUPAN is available on Zenodo with DOI 10.5281/zenodo.2593453 [42]. The raw sequencing data of this paper have been deposited in the European Genome-phenome Archive (https://www.ebi.ac.uk/ega/) under accession EGAS00001003657 [43]. The raw data and assembled contigs of 185 newly sequenced Han Chinese have also been deposited at http://cgm.sjtu.edu.cn/hupan/ and NODE database (http://www.biosino.org/node) with the accession OEP000301 [44]. The data sets of 90 assembled Han Chinese genomes were downloaded from http://gigadb.org/dataset/100302. The non-reference sequences and novel predicted gene sequences from 275 Han Chinese individuals also have been deposited at http://cgm.sjtu.edu.cn/hupan/ and NODE database (http://www.biosino.org/node) with the accession OEP000301 [44]. The novel sequences of hs38d1 [9] were downloaded from NCBI with accession number GCA_000786075.2. The six individual assembled genomes were also downloaded from NCBI. The six primate reference genomes were downloaded from NCBI with accession numbers GCA_000001515.5 (chimpanzee [45]), GCA_000151905.3 (gorilla [46]), GCF_000258655.2 (bonobo [47]), GCA_002880775.3 (orangutan [48]), GCA_000772875.3 (rhesus [49]), and GCF_000264685.3 (baboon [50]). The pan-genome of 910 Africans [20] were downloaded from NCBI under accession PDBU01000000.

## Abbreviations

APG: African pan-genome
INDEL: Insertions and deletions
ORF: Open reading frame
PAV: Gene presence-absence variation
SNV: Single nucleotide variation
SV: Structural variation
