# Supplementary material for: HUPAN: a pan-genome analysis pipeline for human genomes
Source: Genome Biol. 2019 Jul 31;20:149. doi: 10.1186/s13059-019-1751-y (PMC6670167; doi:10.1186/s13059-019-1751-y)
Supplement: Supplementary file 1 — This file contains supplementary methods (section 1), supplementary figures (section 2), and supplementary tables (section 3). (DOC 3790 kb) [file 13059_2019_1751_MOESM1_ESM.doc]

Additional Information for the paper

**HUPAN: A Pan-genome Analysis Pipeline for Human Genomes**

Zhongqu Duan^1,2^, Yuyang Qiao^1^, Jinyuan Lu^1^, Huimin Lu^1^, Wenmin Zhang^1^, Fazhe Yan^1^, Chen Sun^1^, Zhiqiang Hu^1^, Zhen Zhang^3^, Guichao Li^3^, Hongzhuan Chen^4^, Zhen Xiang^5^, Zhenggang Zhu^5^, Hongyu Zhao^2,6^, Yingyan Yu^5*^, Chaochun Wei^1,2,7*^

^1^ School of Life Sciences and Biotechnology, Shanghai Jiao Tong University, 800 Dongchuan Road, Shanghai 200240, China

^2^ SJTU-Yale Joint Center for Biostatistics and Data Science, Shanghai Jiao Tong University, 800 Dongchuan Road, Shanghai 200240, China

^3^ Department of Radiation Oncology and Department of Oncology, Shanghai Medical College, Fudan University Shanghai Cancer Center, Shanghai, PR China，270 Dong An Road, Shanghai 200032, China

^4^ Department of Pharmacology, Shanghai Key Lab. For Translational Medicine, Shanghai Jiao Tong University School of Medicine, 227 South Chongqing Road, Shanghai 200025, China

^5^ Department of Surgery, Ruijin Hospital, Shanghai Key Laboratory for Gastric Neoplasms, Shanghai Jiao Tong University School of Medicine, 197 Ruijin Road, Shanghai 200025, China

^6^ Department of Biostatistics, Yale University, 60 College Street, New Haven, CT 06520, USA

^7^ Shanghai Center for Bioinformation Technology, 1278 Keyuan Road, Pudong District, Shanghai 201203, China

*To whom correspondence should be addressed

**Section 1: Supplementary methods**

# 1.1 Sample preparation and sequencing

Non-neoplastic gastric genome DNA from 185 patients diagnosed with gastric cancer at Ruijin hospital (n=139) and Fudan University Shanghai Cancer Center (n=46) (patients provided their informed consent for use of sample for research) were collected and frozen at -80°C until use. Whole-genome sequencing was performed. Libraries were constructed with ~400-bp insert length, and paired-end 150-bp reads were sequenced on an Illumina HiSeq X10 sequencer. The average sequencing coverage for each individual was 30-fold.

# 1.2 Comparison of de novo assembly using all raw reads and unmapped reads

De novo assembly can help detect genome sequences not present in the current human reference genome. However, due to the large genome size of human, individual genome assembly using all reads needs a huge size of memory. Another strategy is to assembly unmapped reads instead of all reads [1, 2]. In detail, the raw reads are first mapped to the human reference genome and then the unmapped reads are extracted to conduct de novo assembly. This approach could significantly reduce both time and memory. However, the accuracy of this assemble strategy is unknown. We evaluated the performance of these two district assembly strategies on simulated data. Firstly, we simulated a 30-fold depth sequencing data set of 150 bp paired-end pseudo reads with an average insert size of 400 bp by the software NeSSM [3] based on the GRCh38 primary assembly sequences (including 22 autosomes and two sex chromosomes). Then we assembled all reads into contigs by SGA [4] and SOAPdenovo2 [5]. Alternatively, we considered the sequences of chromosome 22 as non-reference sequences and the sequences of rest 23 chromosomes as reference genome. All reads were mapped to the newly constructed reference genome by Bowtie2 [6] with default parameters. The unmapped reads were extracted by Samtools [7] and were assembled by SGA, SOAPDenovo2, MIRA [8] and MiSuRCA [9]. Finally, all the assembled genomes were assessed with the newly constructed reference genome by QUAST [10], which is a tool to evaluate genome assemblies by aligning to reference genome and output various metrics, such as the N50 value, the number of misassembled and unaligned contigs and their lengths. And the contigs that could not be aligned to the newly constructed reference genome were extracted to be assessed based on chromosome 22. The results showed that the approach of only assembling unmapped reads may underestimate the size of non-reference sequences and produce more misassembled sequences, regardless of assembly tools (**Table S1**).

# 1.3 Comparison of de novo assembly software

SOAPdenovo2, ABYSS/ABYSS2 [11, 12] and SGA are among the most popular software for de novo assemble a large genome based on its next generation sequencing data. In EUPAN [13], a “linearK” model that iteratively uses SOAPdenovo2 [5] was applied to choose the “k-mer” for the best assembly result. However, for a human genome, huge memory was required for the depth of 30-fold sequencing data, and it could be prohibitively expensive to deal with a large number of human genomes. The SGA algorithm constructs the FM-index from the preprocessed reads that have been filtered or trimmed for multiple low-quality or ambiguous base calls, corrects base-calling errors using *k*-mer frequencies, re-indexes the corrected reads, removes duplicated sequences and filters out low-quality reads to build a string graph. Finally, the string graph is used to assembled into contigs [4]. We did not use the ABYSS/ABYSS2 because their assemble contigs contain “N”, which may affect the subsequent analysis. Another whole-genome short-read assembler ALLPATHS-LG [14] requires a minimum of two paired-end libraries. Recently, several memory-free assembly software such as BCALM 2 [15] and Minia [16] were available, but their performances were unsatisfactory on real data.

There are several series steps in SGA. The performances of SGA with different settings of parameters were compared with SOAPdenov2 on simulated sequencing data, and the optimal parameters for SGA were determined to be “sga correct -k 55/sga fm-merge –m 65/sga overlap –m 65/sga assemble -m 91 -l 160” (**Table S2)**. In detail, we first conducted *de novo* assembly by SOAPDenove2 and SGA with different parameters on the simulated data set, which was described in section 2. Then the assembly contigs were evaluated to obtain misassembled contigs and unaligned contigs). We choose the parameters with minimum lengths of misassembled contigs and unaligned contigs (**Table S3)**. Finally, the optimal parameters were used to *de novo* assemble each individual to obtain contigs. For the assembled genome size, we could observe two separated classes showed in the **Fig. S1** due to the absence of Y chromosome in female individuals.

We also compared the performances of SOAPdenovo2 and SGA with the optimal parameters on an individual genome (GCH1N00001G) and the results showed the assembled genome size of contigs from SGA was slightly larger than that of SOAPdenovo2, and the N50 of SGA was better (**Table S4**). Although the run time required by SGA was about three times more than that of SOAPdenovo2, the maximum memory utilized in SGA was only one ninth of that of SOAPdenovo2. Low memory consumption makes it possible to run de novo assemble of a deep sequencing human genome in a commodity bioinformatics compute cluster. We were able to assemble the 185 individual deep sequencing genomes in a large scale parallel computing.

# 1.4 Comparison of different strategies to extract non-reference sequences

In order to build the pan-genome sequences, the sequences missing in the reference genome were collected from individual assemblies. However, it is time-consuming and requires a large size of memory to obtain non-reference sequences for the human genomes using EUPAN pipeline (**Main Text Table 1**). Thus, we proposed a two-step strategy: discarding the contigs highly similar with the reference genome followed by the EUPAN strategy to extract non-reference sequence step. In detail, we firstly aligned the assembled contigs to GRCh38 primary assembly sequences by MUMmer package [17] and removed any contigs aligned to the primary assembly sequences with ≥ 95% identity and ≥ 95% of contig length. The remaining contigs were evaluated by QUAST to obtain non-reference sequences. QUAST in EUPAN and the MUMMER step in HUPAN both use the nucmer tool to align sequences to reference genome, but with different parameter setting: QUAST run the nucmer command with the parameter “-maxmatch -c 65 -l 65” and the MUMMER step with the default parameter (-c 65 -l 20).

In comparison, we also used a popular short reads aligner BWA MEM [18] to remove the highly similar contigs according to the CIGAR of alignment result (contigs with the percentage of “M” above 99% is removed). The results showed that combining MUMmer with QUAST could greatly reduce the computing time and memory, while the total number and length of unaligned contigs were almost no difference comparing with running QUAST directly (**Fig. S**2). Thus we proposed the combination strategy to collect the unaligned contigs from each individual in HUPAN.

# 1.5 Removing redundant sequences and contaminated sequences

Before constructing pan-genome, the redundant non-reference sequences among multiple individuals and the potential contaminated sequences should be identified and discarded. We proposed the software CHDIT [19] to remove redundancy of non-reference sequences at the cutoff of 90% identity with parameters “-i input.fa -o output.fa -c 0.9 -T 16 -M 50000”. To exclude the non-human sequences, we aligned the sequences to the NCBI’s non-redundant nucleotide database (30th August, 2017) by NCBI-blast 2.6.0+ [20] with parameters “-out out.blast -max_target_seqs 1 -outfmt 7 -num_threads 16”. According to the taxonomic classification of each sequence, we classified the sequences into five groups: (1) microorganisms, including bacteria, fungi, archaea and viruses; (2) human; (3) non-human primate organisms; (4) non-primate eukaryotes, including plants and non-primate animals; and (5) no alignment to NT database (**Fig. S3**). The sequences classified into (1) and (4) were considered as potential contaminants and were removed in subsequent analysis.

We first merged the non-reference sequences from 185 individuals to obtain 956 Mb fully unaligned sequences as well as 1,047 Mb partially unaligned sequences. After removing redundancy, ~52.9 Mb fully unaligned sequences and ~46.8 Mb partially unaligned sequences were left. Then we classified these sequences according to the taxonomic classification. We altered the thresholds of aligned length and identity and the total lengths of non-reference sequences changed slightly (**Fig. S5**). These results suggest that varying the aligned length and identity thresholds would not have a large influence on the length of novel sequences. In order to reduce the false positive rate, we recommend removing the non-human sequences as much as possible. If a contig aligns to a sequence in NCBI’s non-redundant nucleotide database with aligned length >100 bp and identity >60%, this contig is regarded as from the source species of this sequence. We observed that more than 20 Mb sequences were classified into microorganisms in fully unaligned sequences (**Fig. S5a**). Majority of the partially unaligned contigs were classified into human and other primate (**Fig. S5b**), indicating these sequences were from human genomes.

Alternatively, in order to obtain non-reference sequences from each individual, we calculated the average length of non-reference sequences after removing non-human sequences. In a few individuals, the total length of fully unaligned sequences was significantly larger than the average value and these may due to microbial contaminations (**Fig. S3**), especially the bacterium *Helicobacter pylori*, one majority infectious agent associated with gastric diseases (**Fig. S4**). After removing the sequences labeled as microorganisms and non-primate eukaryotes, the length distribution of individual non-reference sequences was more stable (**Fig. S3**). In addition, we observed that most of non-reference sequences was labeled as human (**Fig. S3**).

We observed that the order of removing contaminated sequences and removing redundant sequences had little impact on the total length of non-reference sequence (**Fig. S6**). Taking the computing time into consideration, we proposed to run the CDHIT first followed by BLAST.

# 1.6 The coverage and frequency of non-reference sequences across individuals

With the available of mapped reads on pan-genome (**Main text**), we could calculate the coverage and frequency of non-reference sequences across 185 individuals. We ran “samtools depth” to compute the depth of all positions for each sequence and calculated the coverage of all non-reference sequences resulted by total mapped bases divided into the total length of the non-reference sequences for each individual. The results suggested the average coverage was 28.96 across 185 individuals. Considering the average sequencing depth of 30-fold across the whole genome, we could draw the conclusion that the coverage across the non-reference sequences is comparable to the coverage across the whole genome.

We then determined the presence absence profile of each sequence. We defined a sequence as present in an individual if over 95% of the sequence is covered by reads, otherwise, the sequence was defined as absence (**Fig. S7**). In total, 6,547 (22.90%) of 28,588 non-reference sequences were presence in all 185 individuals; more than two-third (19,224) of these sequences were presence in at least 100 individuals.

# 1.7 Gene prediction on non-reference sequences

Protein-coding genes on non-reference sequences were predicted using MAKER [21] combing *ab initio* predictions, expression and protein evidences. In detail, RepeatMasker ([http://www.repeatmasker.org](http://www.repeatmasker.org/)) was first run to mask low-complexity repeats. The human gene models were predicted by two *ab initio* predictors, SNAP [22] and AUGUSTUs [23] implemented in MAKER, with default parameters. About 5.2 Gb (8,705,100 entries) human expressed sequences tags (ESTs) and 490 Mb (1,260,728 entries) protein sequences were downloaded from GenBank (12 August 2018). After removing redundancy by CDHIT with the identity of 90%, 2,914, 845 entries of 1.9 Gb EST sequences and 131,272 entries of 48 Mb protein sequences were left. In addition, 90 Mb non-redundant RNA data were obtained from 90 patients diagnosed with gastric cancer. The non-redundant EST sequences, RNA data and protein sequences were aligned to the non-reference sequences with BLASTP or BLASTN. The sequence identified by BLAST around splice sites was realigned by Exonerate [24] to obtain more informative alignments. Finally, the ab initio predictions with RNA and protein evidence were combined and refined by EvidenceModeller [25]. We further filtered the novel predicted genes based on the following stringent criteria:

1. The length of novel genes should be longer than 100 bp;
2. The remaining genes were run by CD-HIT to remove the redundant gene sequences at the identity threshold of 80%, based on the hypothesis that if the identity of two gene sequences is more than 80%, these two genes are considered homology;
3. The remaining gene sequences were aligned to GRCh38 primary genome sequences by BLASTN and the identity (alignment length / gene length) of the best hit should be lower than 50%;
4. The transcription sequences of the remaining genes were aligned to GRCh38 reference transcription sequences by BLASTN and the identity (alignment length / transcription length) of the best hit should be lower than 50%;
5. Defined the full-length gene by determining whether the first three bases of the transcription sequences of novel gene were start codon (ATG) and the last three bases were stop codon (TAG/TAA/TGA).
6. If more than half of the gene sequences were determined into the repeat components by RepeatMasker, the genes were removed.

After the above filtering steps, there were 167 full-length novel genes retained (**Table S5, Fig. S8)**. About one-third (56/167) of the novel genes were shorter than 500 bases, 141 (84.43%) genes were shorter than 1,000 bases. More than half (90/167) of novel genes had two CDS exons and 36 (21.56%) genes had more than two CDS exons.

# 1.8 Validation of novel predicted genes

We verified the novel genes with RNA sequencing data sets. Ninety of the 185 deep sequencing Han Chinese genomes also had conducted on RNA sequencing and obtained 9.48 billion paired-end reads with 100 bp length. In addition, we downloaded 1,001 RNA data, including 646 pair-end RNAseq datasets and 355 single-end RNAseq samples, from SRA database (ERP023007, ERP107734, SRP098916, SRP100417, SRP102685, SRP110699, SRP111343, SRP118127, SRP119923, SRP135952, SRP133891). The reads were mapped to the transcripts of novel predicted genes by hisat2 [26] with default parameters. The coverage of each transcript was calculated and we altered the defined coverage (80%, 85%, 90% and 95%) to determine whether the transcript was present in a certain sample. And if the transcript presented at least one sample, we considered the transcript was validated.

# 1.9 Appling HUPAN in 90 Han Chinese genomes

With the available of assembled result from 90 unrelated individuals of Chinese ancestry, which consisting of 45 Northern Han Chinese and 45 Southern Han Chinese samples [27], we can perform pan-genome analysis to detect the non-reference sequences. Firstly, the assembled scaffolds were downloaded from <http://gigadb.org/dataset/100302>. The scaffolds were split into scaftigs at the position of “N”. Then the scaftigs of each individual were aligned to the GRCh38 primary genome sequences by MUMmer to obtain candidate unaligned sequences. The candidate unaligned sequences were accessed by QUAST to extract non-reference sequences. All the non-reference sequences from 90 individuals were merged and removed redundant sequences by CDHIT at the minimum identity of 90%. Then the non-redundant sequences were aligned to NT database by BLASTN to remove potential contaminations. The non-reference sequences were used to predict novel genes by MAKER. The novel genes or gene segments were further screened following the six criterions listed in the section “Gene prediction on non-reference sequences”.

In total, there were 10.37 Mb fully unaligned sequences detected in 90 individuals (**Table S6**). The length distribution was similar to that of 185 deep sequencing Han Chinese genomes (**Fig. S10a**). We also explored the similarity among the sequences as well as with the human reference genome using lower identity. The results indicated that parts of sequences were similar to each other and the majority of the sequences cannot map to the reference genome even when the threshold of identity percentage was decreased to 80% (**Fig. S10b&c**). The repeat component analysis showed that about 65% of 10.37 Mb consisting of various types repeat sequences (**Fig. S10d**). Of 10.37 Mb sequences, 96.64% could be aligned to at least one of the ALT sequences and six previously published human genome assemblies [2, 25-29] (**Fig. S10e**, **Table S7**). In total, 79 full-length novel genes were predicted on these 10.37 M non-reference sequences. Thirty-one (46.37%) of the 79 novel predicted genes were shorter than 500 bases, and 68 (86.08%) were shorter than 1 Kb (**Table S8**). More than 63% (50/79) of genes had two or more CDS exons (**Table S8**). More than 60% (48/79) of the full-length novel genes could be validated by RNA-Seq data at 95% coverage of transcripts (**Fig. S12**).

# 1.10 Origin of fully unaligned sequences

To determine the origin of the sequences, we aligned fully unaligned sequences from both 185 newly sequenced and 90 assembled Han Chinese genomes to the available non-human primate reference genomes. Six primates were used to explore the origin of the sequences and their reference genomes were downloaded from NCBI. The GenBank assembly accession or RefSeq assembly accession used in this study were chimpanzee [28] (panTrp5, GCA_000001515.5), gorilla [29] (gorGor4, GCA_000151905.3), bonobo [30] (panPan2, GCF_000258655.2), orangutan [31] (ponAbe3, GCA_002880775.3), rhesus [32] (rheMac8, GCA_000772875.3) and baboon [33] (Panu3, GCF_000264685.3). These sequences were aligned against each of these non-human primate’s reference genomes using nucmer with the parameter “-c 65 -l 65 --maxmatch”. Only sequences that aligned with at least 90% identity and more than 100 bp were considered as real hits. The total length of sequences aligned to non-primate reference genome were decreased along with the increase of evolutionary distance with human in phylogenetic tree. In aggregate, almost half of these sequences had a partial match to at least one primates (**Table S9**).

# 1.11 Comparison with the African pan-genome

Recently, the African pan-genome (APG) from deep sequencing of 910 individuals revealed 296,485,284 bp in 125,715 distinct contigs (called APG contigs) [1]. To underscore the pan-genome between our data sets and African, we firstly collapsed the novel sequences from 185 deep sequencing genome and 90 assembled Han Chinese genomes into a combined data set. Due to the placed sequences both on GRCh38 primary assembly sequences and all patch sequences and alternative loci were not included in final APG contigs, we firstly excluded the aligned sequences of partially unaligned sequences. The remaining novel sequences which longer than 500 bp were merged with the fully unaligned sequences and further remove redundancy. Finally, we obtained a non-redundant novel sequences dataset, which absolutely novel for GRCh38 primary assembly sequences. We then aligned these sequences to the patch sequences and alterative loci from GRCh38.p8 by nucmer with the parameter “-c 65 -l 65 --maxmatch”. In total, 4.08 Mb (12.15%) could align to the additional sequences with ≥ 90% identity and ≥ 80% coverage. The remaining novel sequences were aligned to the novel sequences from the Simons Genome Diversity Project [34] (hs38d1) and African pan-genome (APG) contigs, respectively. All alignments were performed by nucmer with the parameter “-c 65 -l 65 --maxmatch”, and the best record of alignment for each sequence was retained and further filtered with an over identity ≥ 90% that covered ≥ 80% of the sequence. In addition, we counted the number of the non-redundant novel genes after removing redundant genes by CDHIT at the identity threshold of 80%. And the remaining genes were aligned to the patch sequences and alterative loci by BLAST and determined whether they have homologous sequences.

More detailed analysis was conducted by comparing our data set with APG contigs. We reciprocally obtained each APG contig’s alignment of our data set as previously described. At the identity of 90%, we counted the accumulated length of alignment in different coverage ranges (**Fig. S13**). With the criterion of 100% coverage, 9.91 and 8.99 Mb sequences were actually aligned with another for our data sets and APG contigs, respectively (**Fig. S13**), indicated these sequences were common in both Han Chinese individuals and African individuals, but not included in the reference genome. In comparison, several megabases novel sequences have no alignment or only partially aligned with another (**Fig. S13**). Given the amount of our data set only 29.50 Mb, multiple APG contigs could map one sequences in our data set (**Fig. S13**).

# 1.12 Computational resources

HUPAN is implemented in Perl, R and C ++ and designed to conduct hundreds or thousands of individuals in parallel on a computer cluster with LSF/SLURM system or orderly on a single machine. In this manuscript, all the bioinformatics analyses were conducted on High Performance Computing Center (HPCC) at Shanghai Jiao Tong University. The main computational bottleneck of HUPAN pipeline was de novo assembly of nearly 200 individuals. We conducted the assemblies in parallel on 323 supercomputers with eight Cores 2.6 GHz CPUs with 64 GB of memory installed (called CPU nodes), and used the Linux operating system. On average, assembly of one individual took about 70 hours, with 16 threads parallelization, resulting in more than one thousand CPU core hours. All the processes of de novo assembly on simulated data also run on the CPU nodes. De novo assembly with SOAPDenovo2 was run on a machine with the memory size of 2 TB. The process of aligning assembled contigs by MUMmer was run on fat nodes, which has the memory of 256 Gb. All other analyses were run on the CPU node.

**Section 2: Supplementary figures**

**
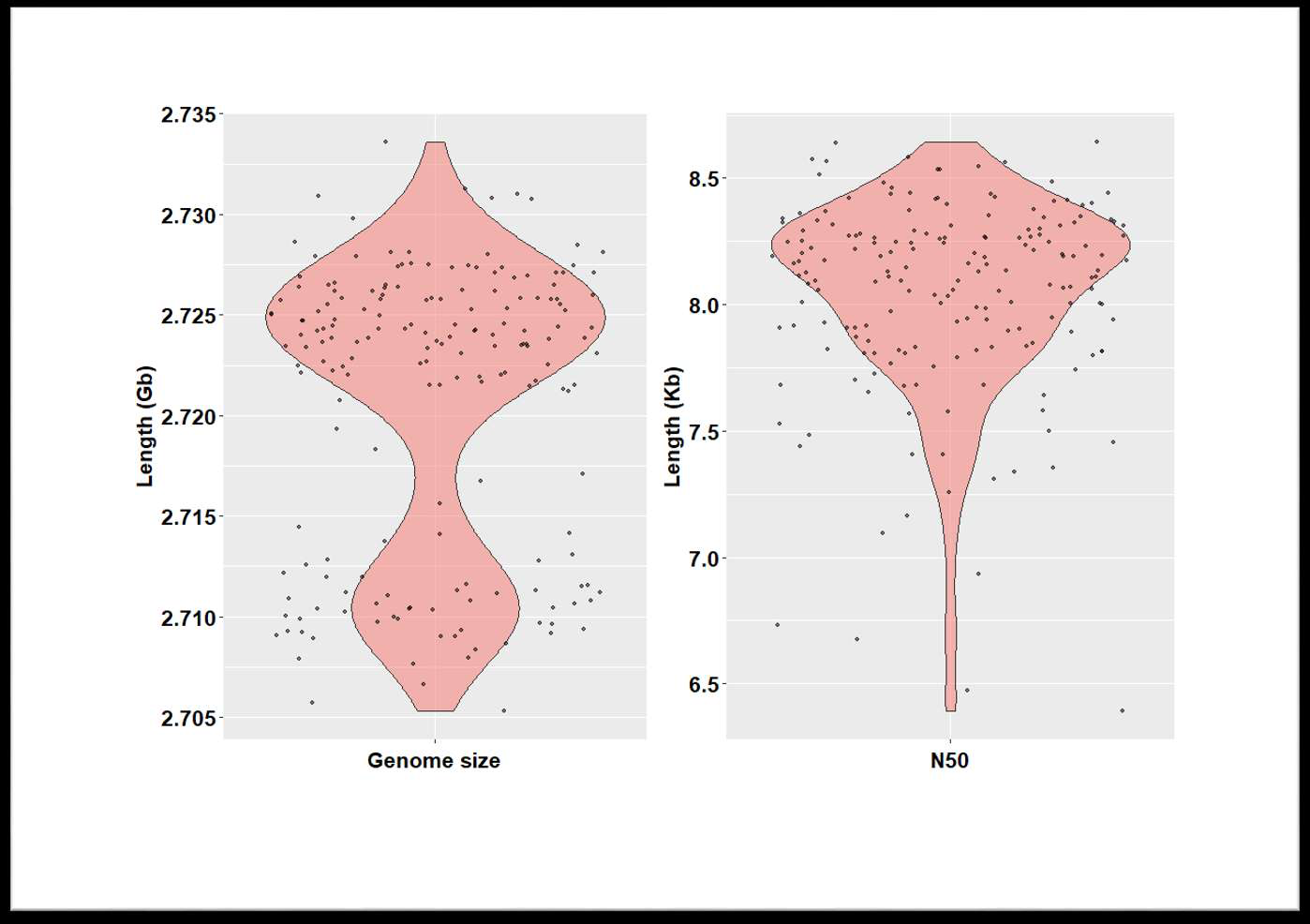
**

**Fig. S1:** Assembled genomes size (Gb) and N50 (Kb) of 185 deep sequencing Han Chinese genomes. The statistical summaries were resulted from the contigs which longer than 500 bp for each individual.


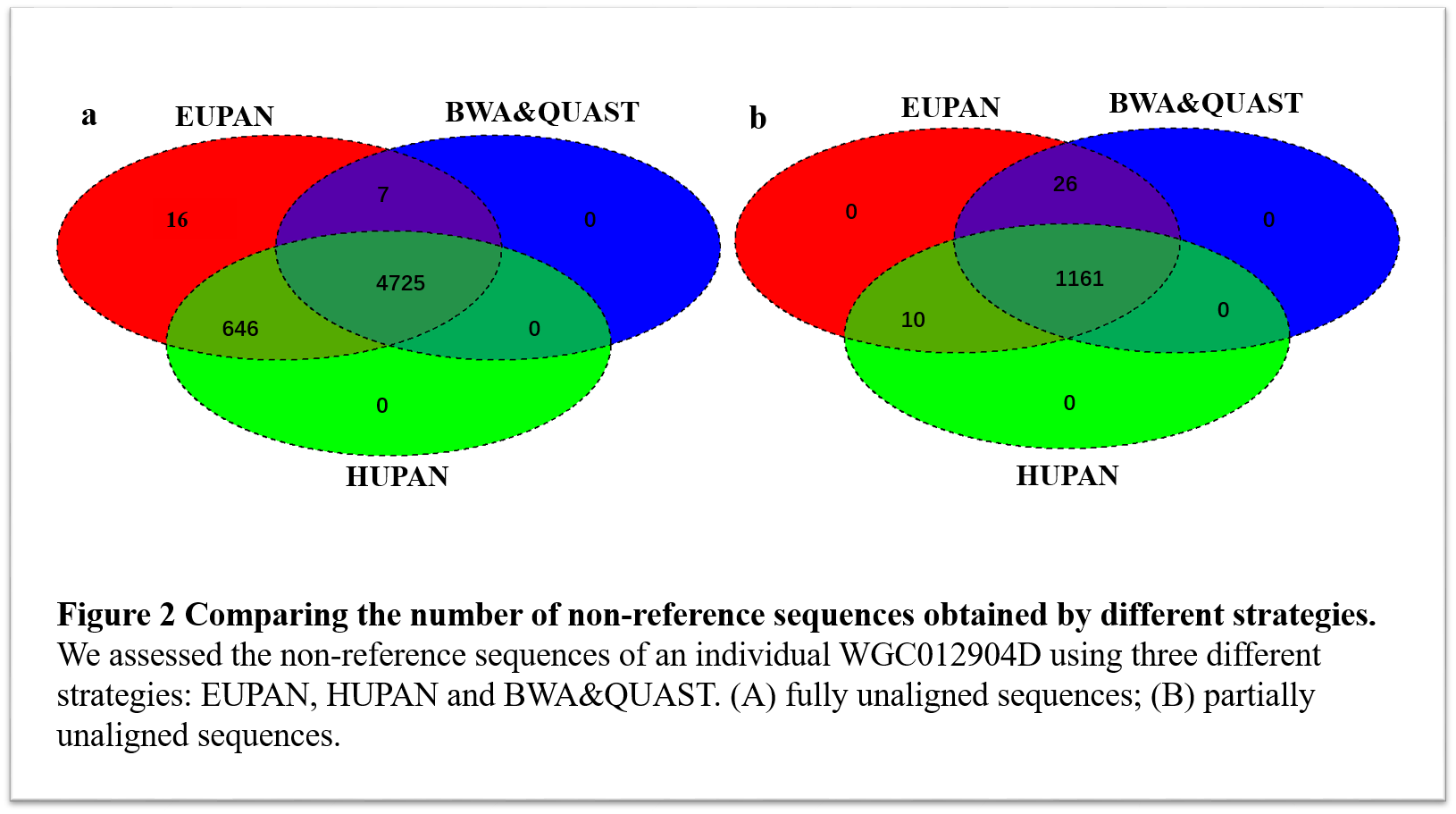


**Fig. S2:** Comparing the number of non-reference sequences obtained by different comparison strategies. (**a**) fully unaligned sequences; (**b**) partially unaligned sequences.

**
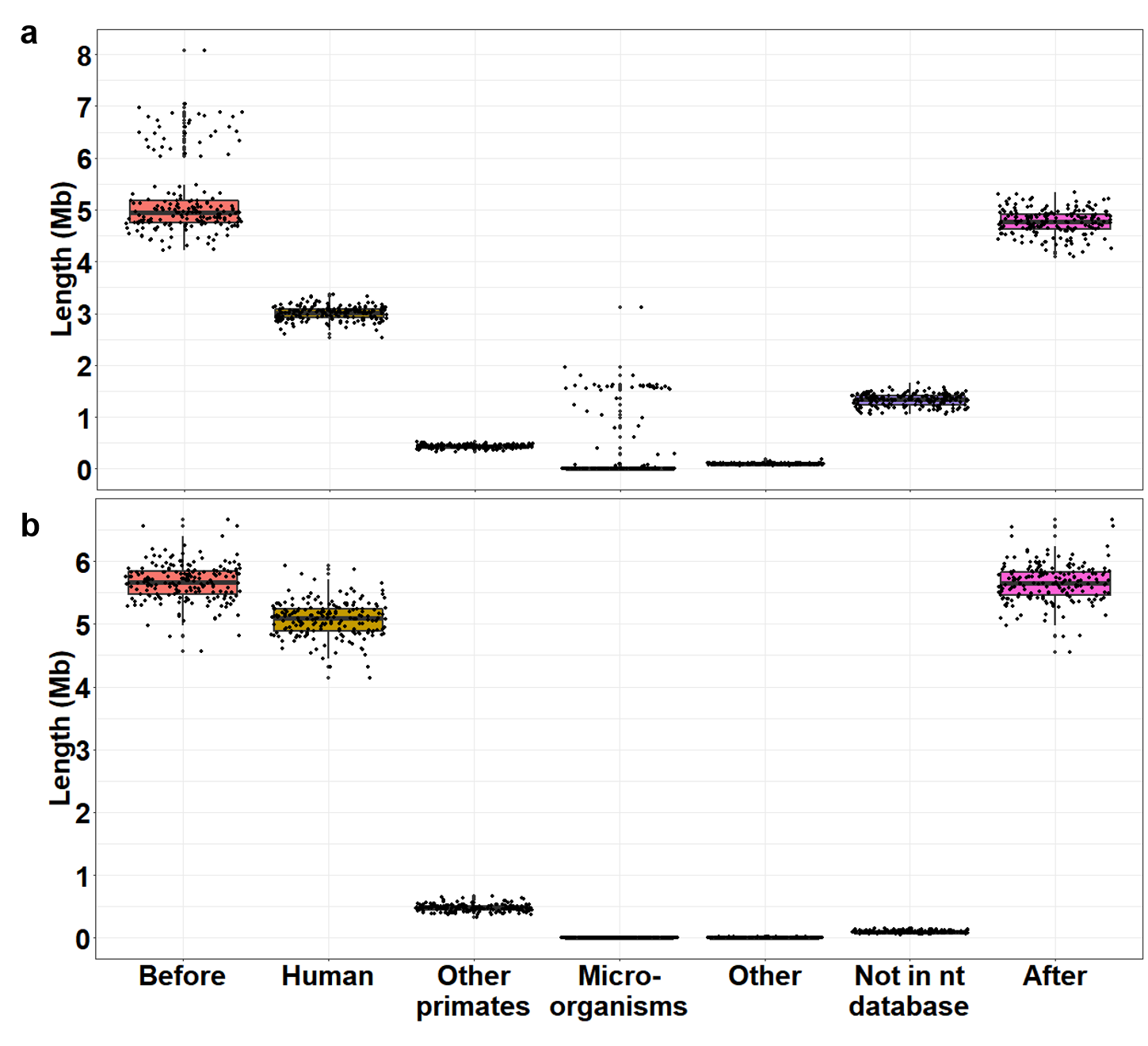
**

**Fig. S3:** The non-reference sequences in 185 deep sequencing Han Chinese genomes. (**a**) Fully unaligned sequence; (**b**) Partially unaligned sequences.

**
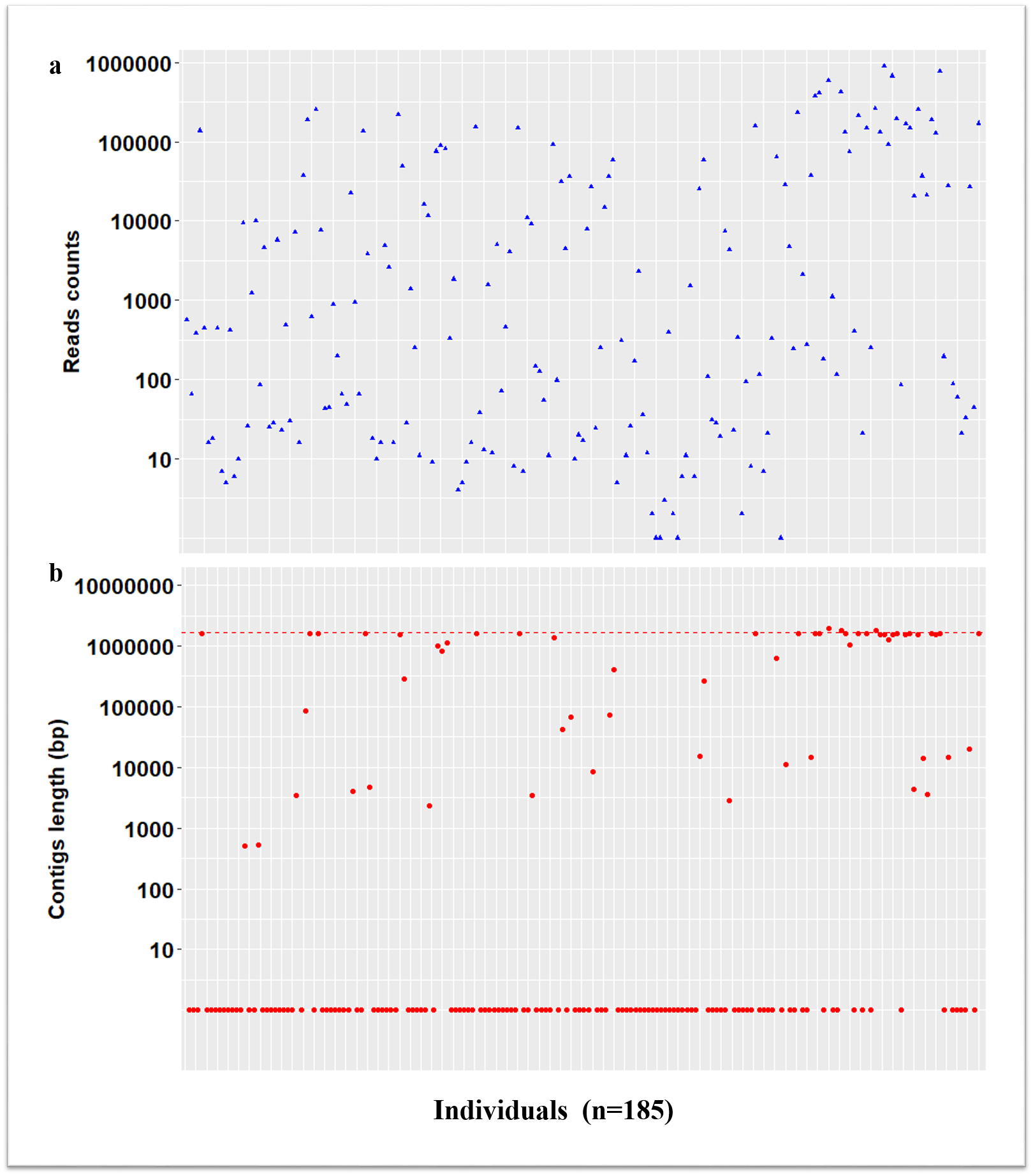
**

**Fig. S4:** The number of reads and total length of contigs (bp) mapped to the bacterium *Helicobacter pylori*. **(a)** The number of reads obtained by mapping the unmapped reads of GRCh38 to the *Helicobacter pylori* reference genome (*Helicobacter pylori* 26695). **(b)** The total length of contigs obtained by mapping the non-reference sequences to the *Helicobacter pylori* reference genome for each individual. The red dashed line indicated that the genome size of Helicobacter pylori reference genome (1.67 Mb).


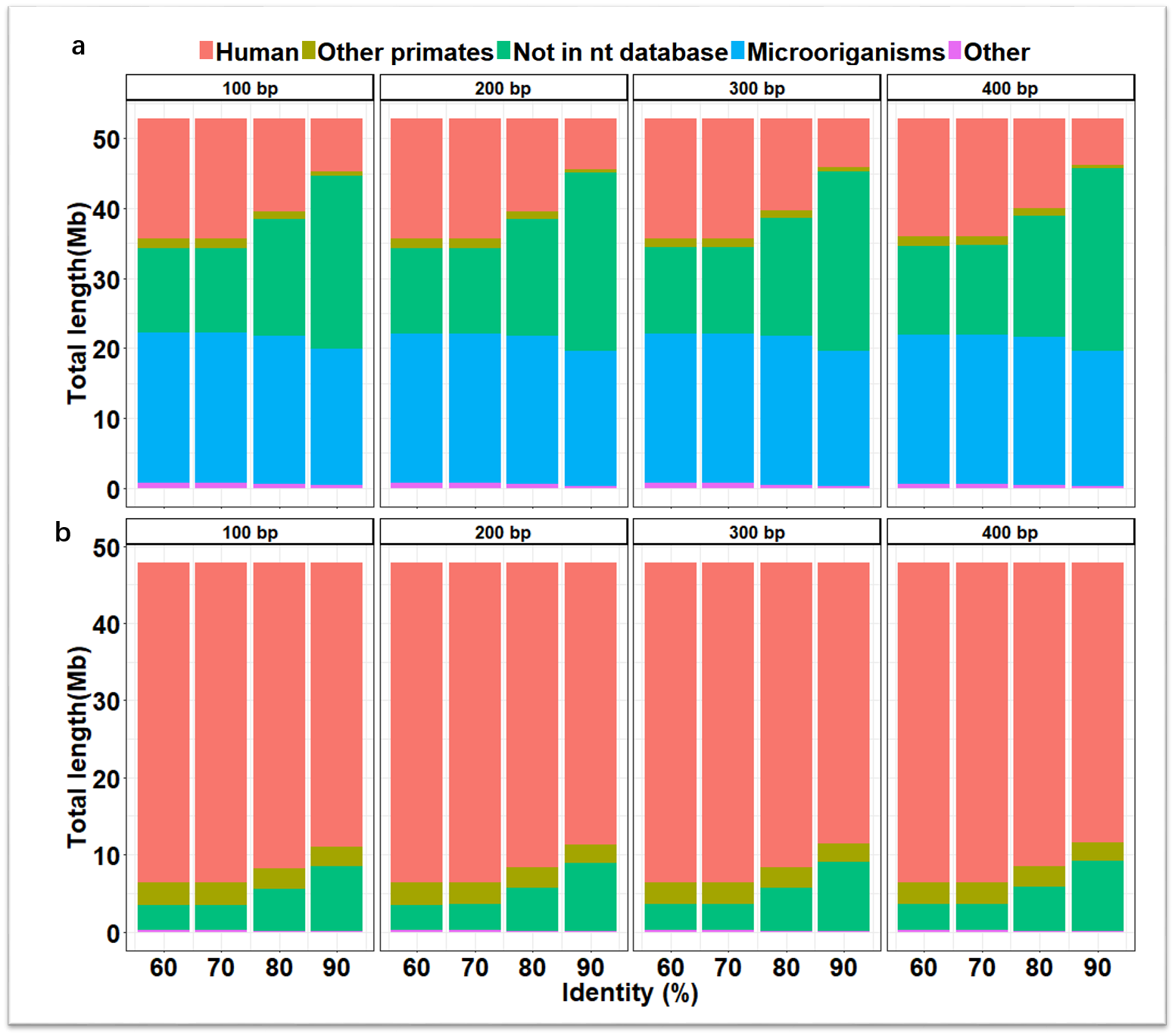


**Fig. S5:** The classification of the non-redundant non-reference sequences under different thresholds of aligned length and identity in 185 deep sequencing Han Chinese genomes. These plots show the total length (Mb) of sequences mapped to different types with different alignment lengths and identities about the fully unaligned sequences (**a**) and the partially unaligned sequences (**b**).

**
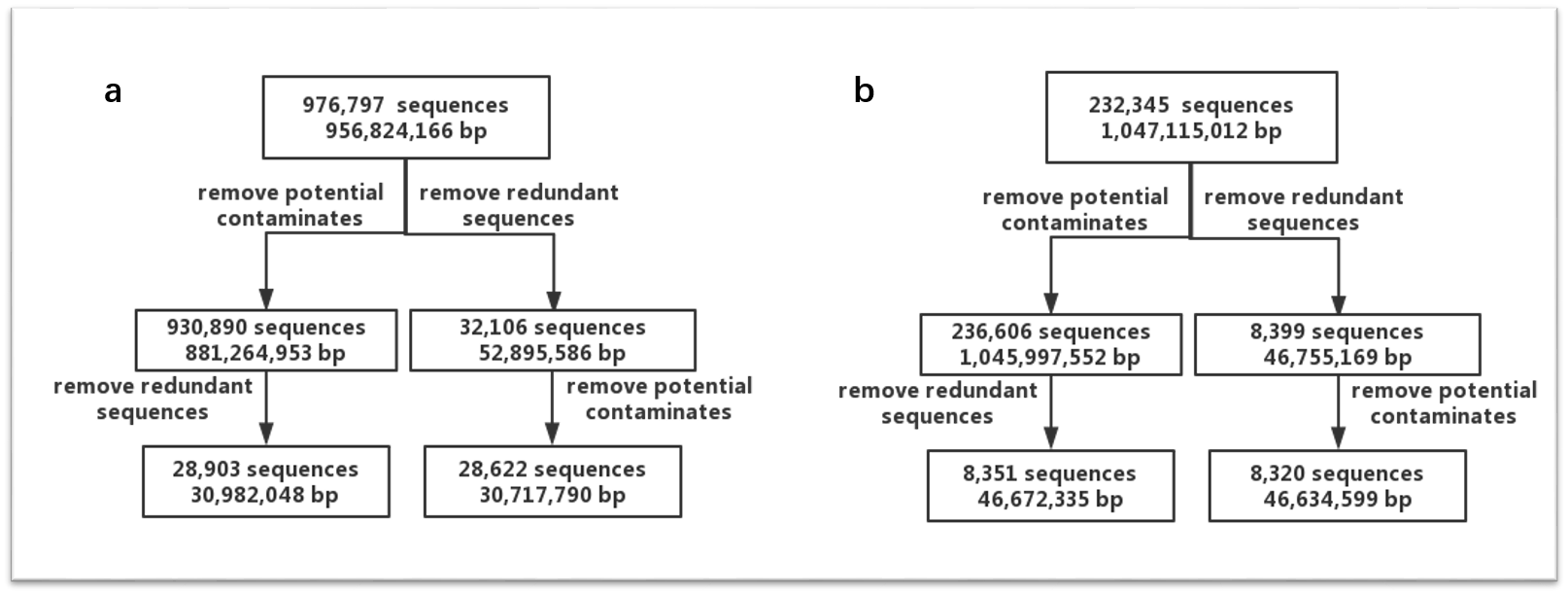
**

**Fig. S6:** The processes of removing redundant sequences and potential contaminations in 185 deep sequencing Han Chinese genomes. **(a)** Fully unaligned sequence; **(b)** The partially unaligned sequences.


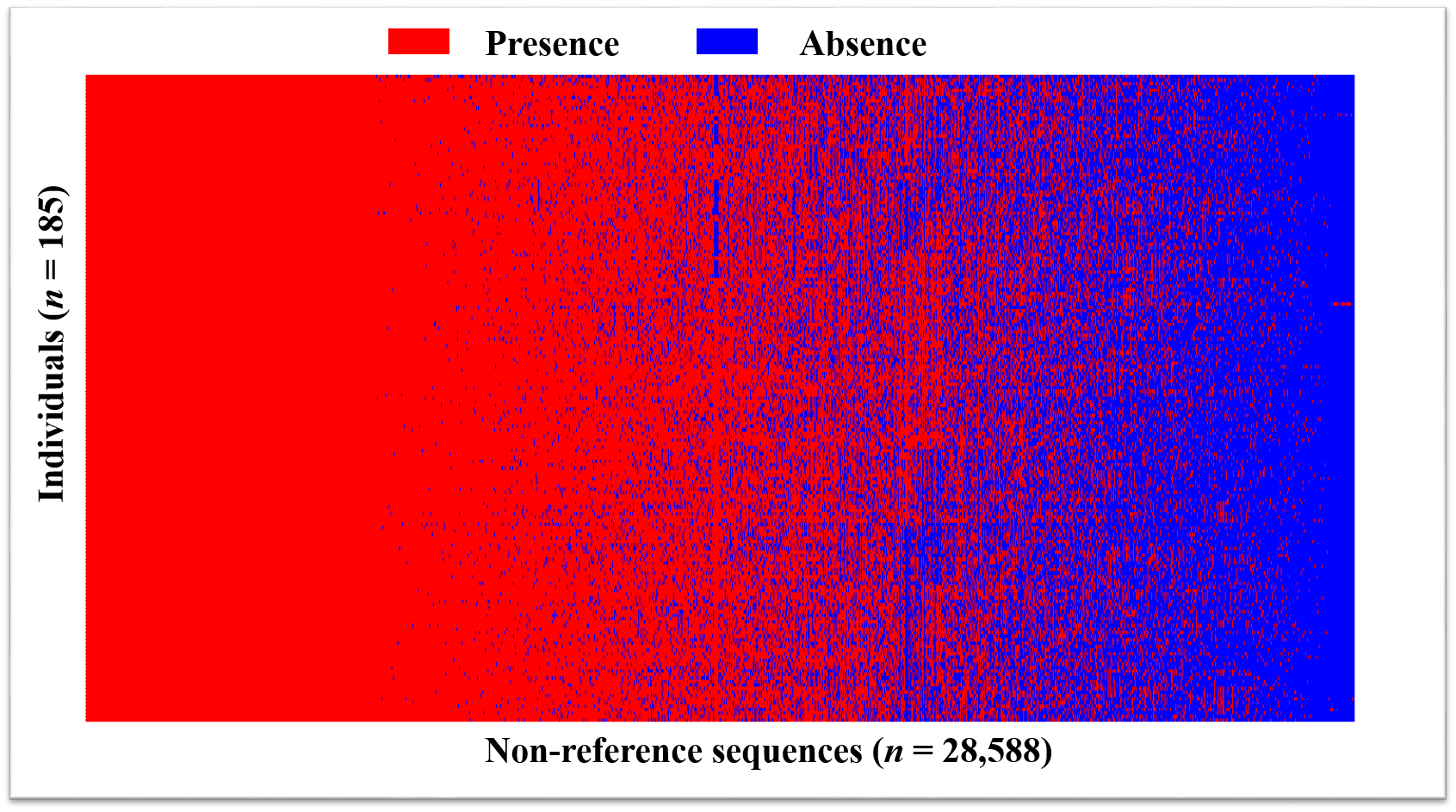


**Fig. S7:** The presence absence profile of non-reference sequences across 185 individuals.

**
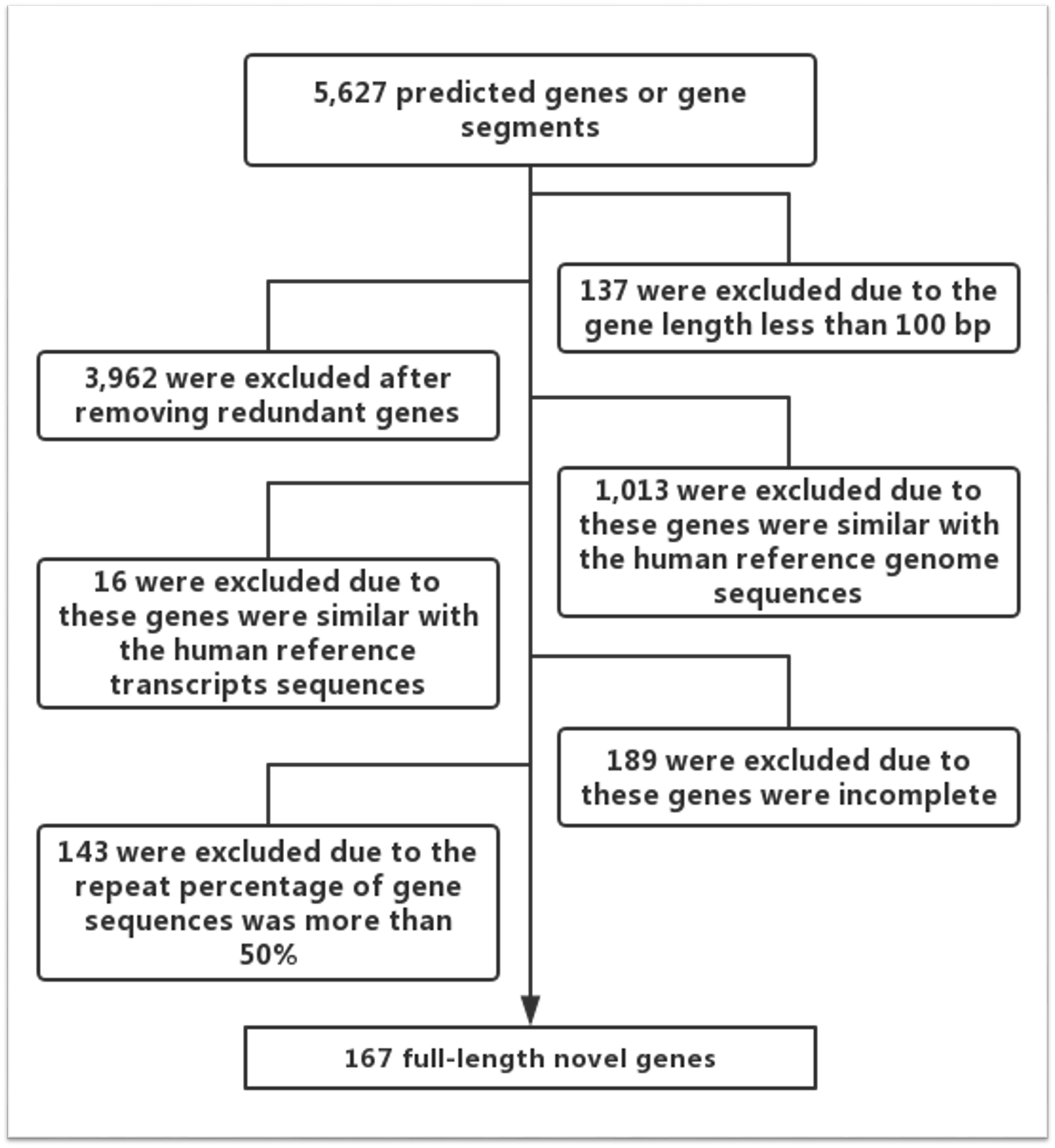
**

**Fig. S8:** The processes of filtering the 5,627 predicted genes or gene segments in 185 deep sequencing Han Chinese genomes.


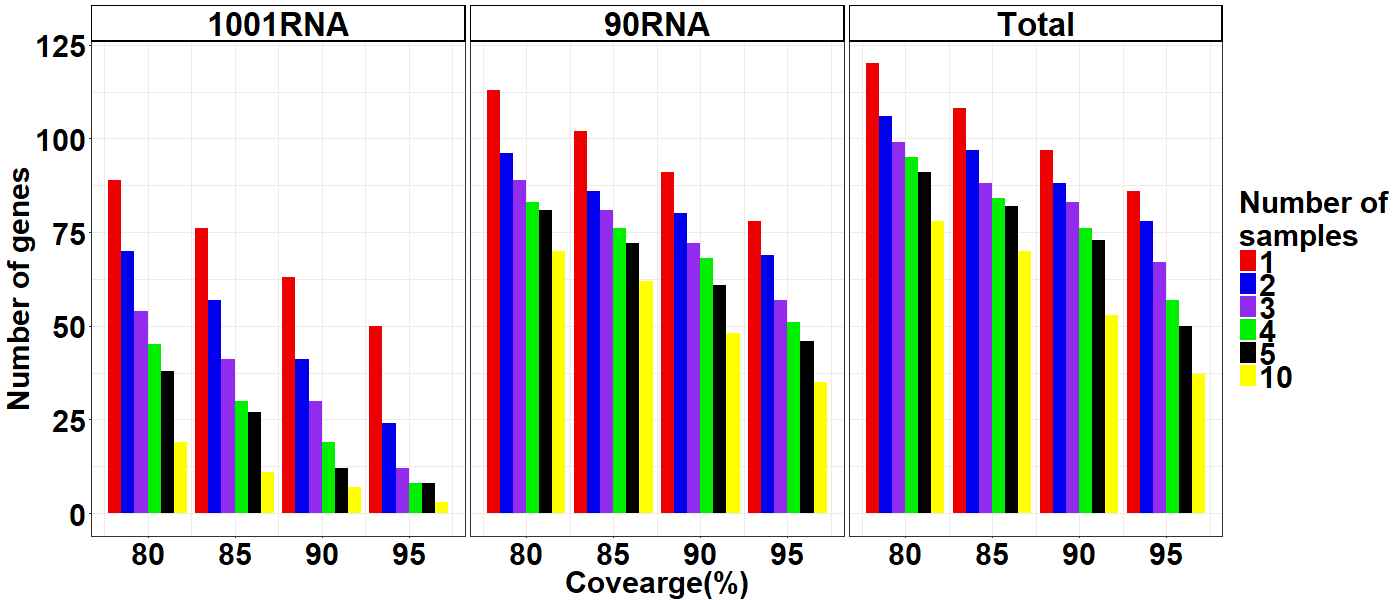


**Fig. S9:** Validation of 167 novel genes predicted from 185 deep sequencing Han Chinese genomes by RNA-seq data. “1000 RNA” 1001 RNA-seq data from public data; “90RNA” was used the 90 RNA-seq data from gastric tissues; and “Total” was the results from combined two data sets. The number of samples indicated the novel gene could validate at last n individual(s), and n=1, 2, 3, 4, 5, 10.

**
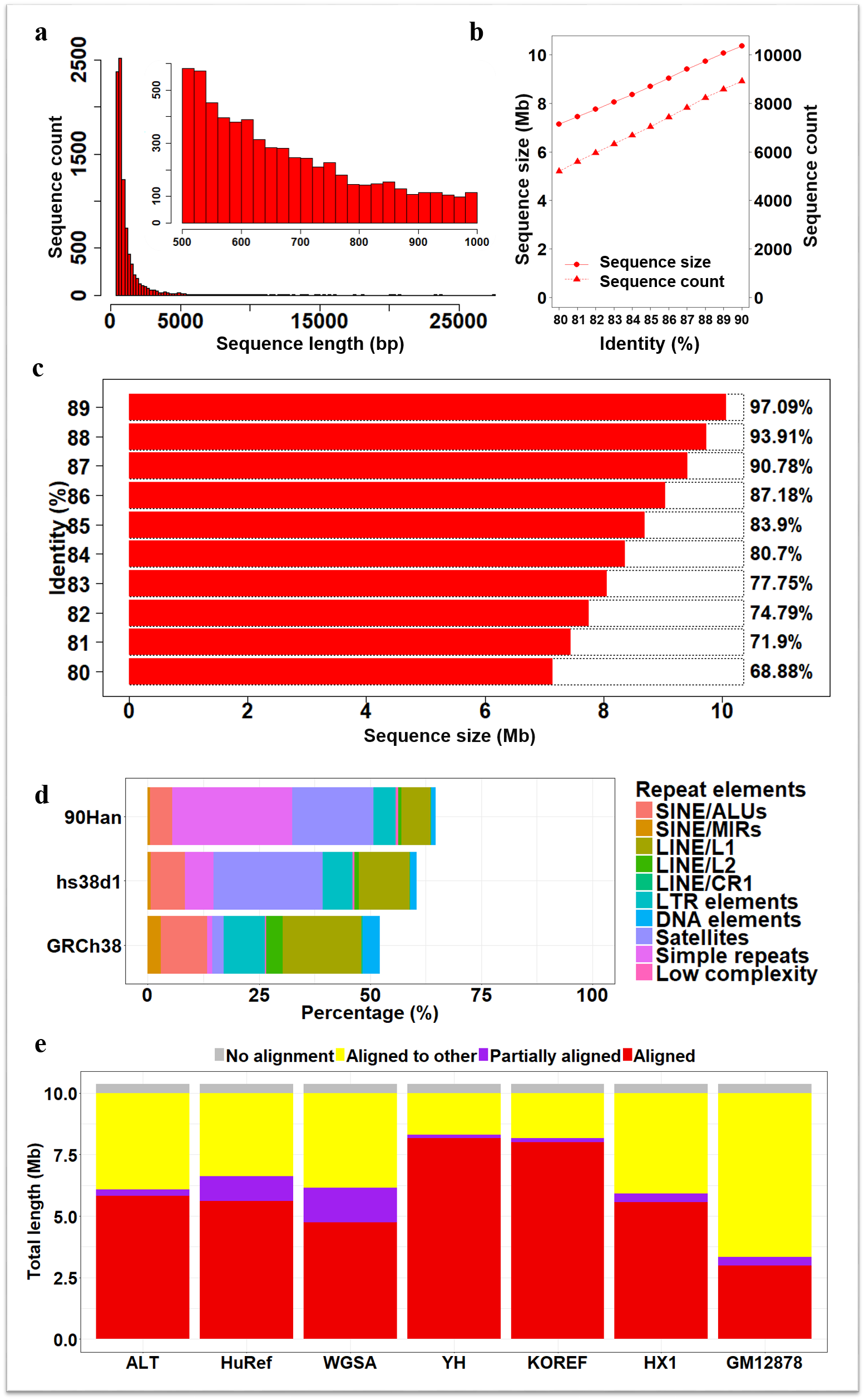
**

**Fig. S10:** Characterization of sequences fully unaligned to the human reference genome in 90 Han Chinese genomes. See note of Fig. 4 in main text.

**
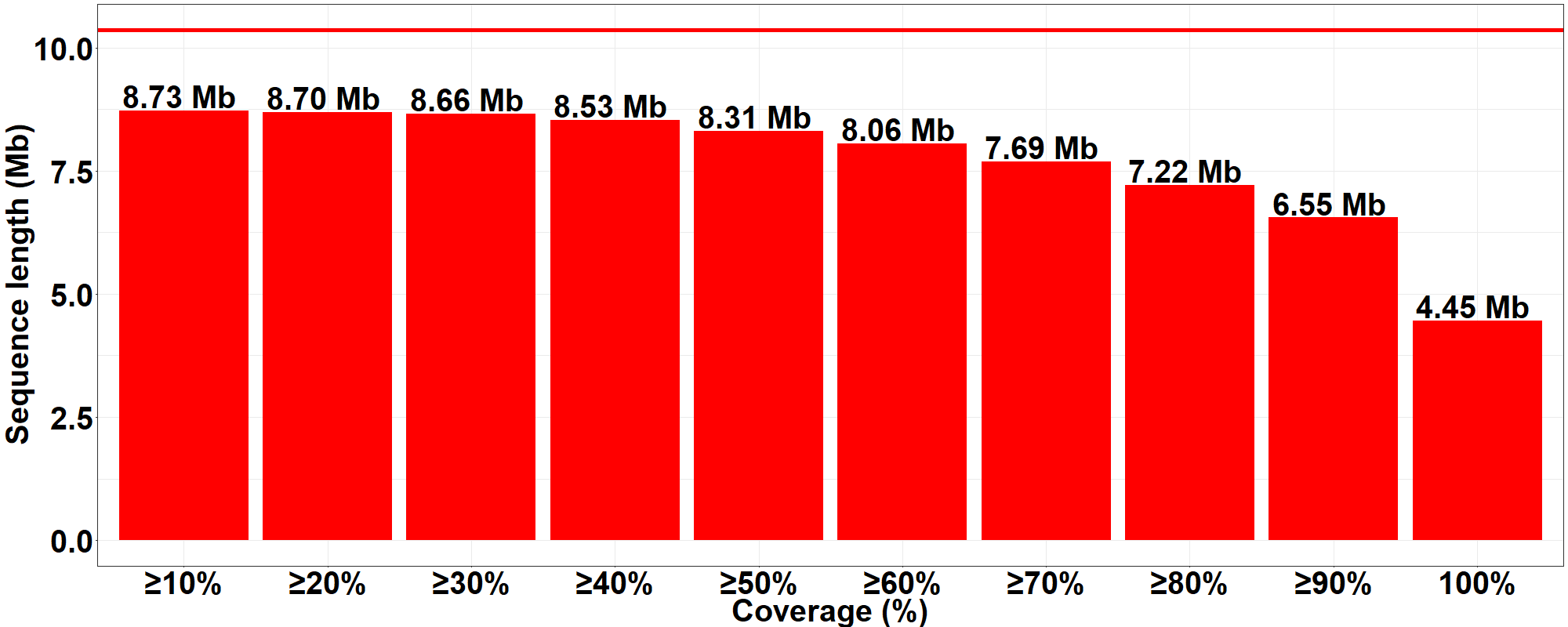
**

**Fig. S11:** Validation of fully unaligned sequences from 90 Han Chinese genomes by 185 deep sequencing Han Chinese genomes with sequence identity ≥ 90% using difference sequence coverages.


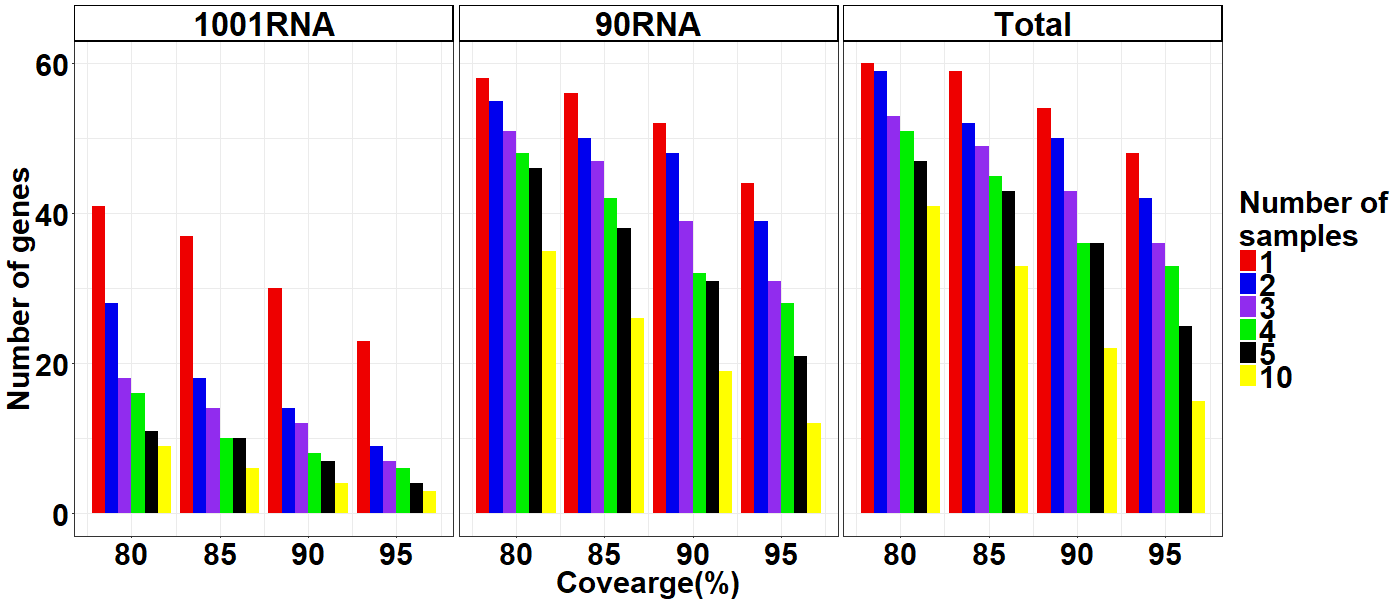


**Fig. S12:** Validation of 79 novel genes predicted from 90 Han Chinese genomes by RNA-seq data.


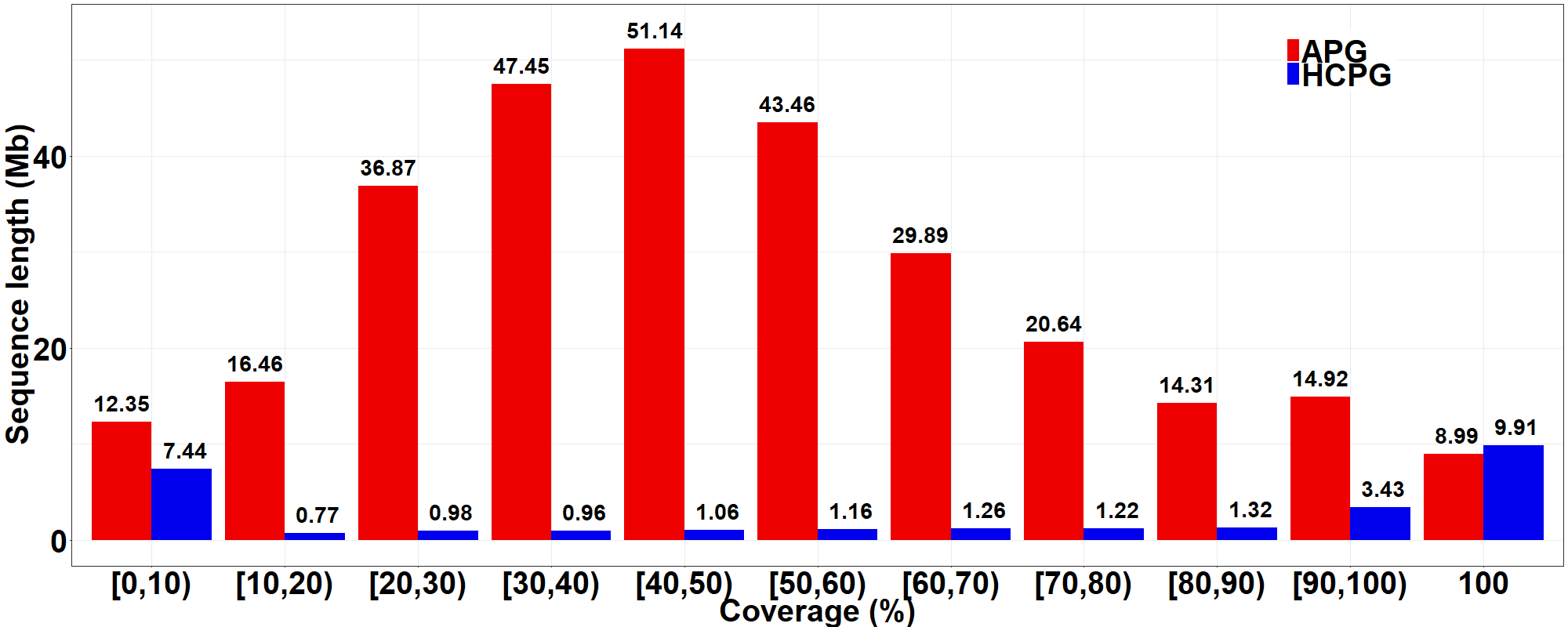


**Fig. S13:** The length distribution of novel sequences from APG and HCPG that could be aligned to each other with sequence identity ≥ 90% using difference sequence coverages. APG: the results of APG aligned to HCPG; HCPG: the results of HCPG aligned to APG.

**Section 3: Supplementary tables**

**Table S1:** Assembled results from de novo assembly using all reads and unmapped reads.

|  | All reads | | |  | | Unmapped reads | | | |  |
| --- | --- | --- | --- | --- | --- | --- | --- | --- | --- | --- |
|  | SGA | SOAPDenovo2 |  | | SGA | | SOAPDenovo2 | MIRA | MaSuRCA | |
| # contigs | 3,403 | 2,834 |  | | 2,959 | | 3,681 | 9,906 | 5,361 | |
| Total length (bp) | 32,224,073 | 32,264,453 |  | | 31,689,216 | | 31,553,001 | 32,059,525 | 28,003,137 | |
| # misassembled contigs | 0 | 0 |  | | 5 | | 3 | 275 | 99 | |
| Misassembled contigs length (bp) | 0 | 0 |  | | 138,574 | | 69,018 | 634,870 | 749,725 | |
| # unaligned contigs | 0 | 0 |  | | 0 | | 0 | 3 | 0 | |
| Unaligned contigs length (bp) | 0 | 0 |  | | 0 | | 0 | 2,340 | 0 | |

**Table S2**: SGA de novo assembly parameters.

| SGA step | Parameters |
| --- | --- |
| preprocess (preprocess fastq files) | --pe-mode 1 |
| index (index preprocessed data) | -a ropebwt –no-reverse –t 16 |
| correct (error correction on preprocessed data) | -k 55 –learn –t 16 |
| index (index error-corrected data) | -a ropebwt –t 16 |
| filter (filter error-corrected data) | -x 2 –t 16 |
| fm-merge (merge reads) | -m 65 –t 16 |
| index (index FM-merged reads) | -d 20000000 –t 16 |
| rmdup (remove duplicates) | -t 16 |
| overlap (construct string graph) | -m 65 –t 16 |
| assemble (contig assemble) | -m 91 –l 160 |

**Table S3**: The assemblies of contigs by SOAPdenovo2 and SGA using simulated data.

| Type | SGA | SOAPdenovo2 |
| --- | --- | --- |
| Total length (bp) | 2,750,400,059 | 2,749,036,269 |
| N50 (bp) | 26,527 | 29,981 |
| # misassembled contigs | 2 | 7 |
| Misassembled contigs length (bp) | 25,320 | 58,363 |
| # Unaligned contigs | 0 | 0 |
| Unaligned contigs length (bp) | 0 | 0 |

**Table S4**: The performances of HUPAN and EUPAN on assembling an individual genome.

| Type | HUPAN | EUPAN |
| --- | --- | --- |
| Assembled genome size (>500bp) (bp) | 2,709,735,693 | 2,664,443,084 |
| N50 (bp) | 8,069 | 6,174 |
| # Misassembled contigs | 1,037 | 1,071 |
| Misassembled contigs length (bp) | 5,483,408 | 5,025,281 |
| # Fully unaligned contigs | 5,371 | 5,922 |
| Fully unaligned sequences length (bp) | 5,000, 779 | 5,509,739 |
| # Partially unaligned contigs | 1,187 | 1299 |
| Partially unaligned sequences length (bp) | 5,435,999 | 5,141,866 |
| Maximum memory (Gb) | 60 | 531 |
| Walk time (thread=16) (hours) | 73.5 | 27 |

**Table S5**: The length distribution and the CDSs number of novel prediction genes detected in 185 deep sequencing Han Chinese genomes.

| Type |  | Number of genes |
| --- | --- | --- |
| Length distribution  (bp) | [100,500] | 56 |
|  | [500,1000] | 85 |
|  | [1000,5000] | 25 |
|  | [5000,10000] | 1 |
| Number of CDS | 1 | 41 |
|  | 2 | 90 |
|  | 3 | 29 |
|  | 4 | 4 |
|  | 5 | 1 |
|  | 9 | 2 |
| Total | | 167 |

**Table S6**: Summary of fully unaligned sequences obtained from 90 Han Chinese genomes.

|  | Number | Total length (bp) |
| --- | --- | --- |
| Merge 90 individuals | 285,673 | 318,657,773 |
| Remove redundant sequence | 9,390 | 10,972,868 |
| Human  Other primates | 5,758  847 | 7,353,460  905,231 |
| No alignment to NT database | 2,310 | 2,112,223 |
| Microorganisms | 344 | 404,687 |
| Non-primate | 131 | 197,267 |
| Non-reference sequences | 8,915 | 10,370,914 |

**Table S7**: Validation of fully unaligned sequences obtained from 90 Han Chinese genomes by aligning to other available human sequences (> 90% identity).

| Assembled genome | Alignment (bp) | Partially unaligned (bp) | Fully unaligned (bp) |
| --- | --- | --- | --- |
| ALT | 5,819,212 | 259,716 | 4,291,986 |
| HuRef | 5,600,212 | 1,020,861 | 3,749,841 |
| WGSA | 4,744,023 | 1,402,807 | 4,224,084 |
| YH | 8,170,013 | 125,899 | 2,075,002 |
| KOREF | 7,996,161 | 163,887 | 2,210,866 |
| HX1 | 5,558,492 | 354,609 | 4,457,813 |
| GM12878 | 2,982,877 | 345,842 | 7,042,195 |
| Unknown | 370,222 | | |

**Table S8**: The length distribution and the CDSs number of novel prediction genes detected in 90 Han Chinese genomes.

| Type |  | 90Han |
| --- | --- | --- |
| Length distribution  (bp) | [100,500] | 39 |
|  | [500,1000] | 29 |
|  | [1000,5000] | 11 |
|  | [5000,10000] | 0 |
| Number of CDS | 1 | 29 |
|  | 2 | 39 |
|  | 3 | 9 |
|  | 6 | 1 |
|  | 8 | 1 |
| Total | | 79 |

**Table S9**: Fully unaligned sequences identification in non-human primates.

| Origin | 185 newly sequenced genomes | |  | 90 assembled genomes | |
| --- | --- | --- | --- | --- | --- |
|  | Number | Length (bp) |  | Number | Length (bp) |
| Chimpanzee (panTro5) | 8239 | 10691913 |  | 3460 | 4383629 |
| Gorilla (gorGor4) | 7,582 | 8,948,723 |  | 2,757 | 3,634,777 |
| Bonobo (panPan3) | 5,510 | 7,301,524 |  | 2,892 | 3,785,220 |
| Orangutan(ponAbe3) | 3,673 | 4,624,774 |  | 2,349 | 3,243,693 |
| Rhesus (rheMac8) | 1,113 | 1,873,038 |  | 1,047 | 1,816,020 |
| Baboon (Panu3) | 1,021 | 1,693,729 |  | 1,011 | 1,750,074 |
| All non-human primates | 12,387 | 14,827,976 |  | 4,125 | 5,165,706 |
| All sequences | 28,622 | 30,717,790 |  | 8,915 | 10,370,914 |
| Validation ratio | 43.28% | 48.27% |  | 46.27% | 49.81% |

# References

1. Sherman RM, Forman J, Antonescu V, Puiu D, Daya M, Rafaels N, Boorgula MP, Chavan S, Vergara C, Ortega VE, et al: **Assembly of a pan-genome from deep sequencing of 910 humans of African descent.** *Nat Genet* 2018.

2. Faber-Hammond JJ, Brown KH: **Anchored pseudo-de novo assembly of human genomes identifies extensive sequence variation from unmapped sequence reads.** *Hum Genet* 2016, **135:**727-740.

3. Jia B, Xuan LM, Cai KY, Hu ZQ, Ma LX, Wei CC: **NeSSM: A Next-Generation Sequencing Simulator for Metagenomics.** *Plos One* 2013, **8**.

4. Simpson JT, Durbin R: **Efficient de novo assembly of large genomes using compressed data structures.** *Genome Research* 2012, **22:**549-556.

5. Luo R, Liu B, Xie Y, Li Z, Huang W, Yuan J, He G, Chen Y, Pan Q, Liu Y, et al: **SOAPdenovo2: an empirically improved memory-efficient short-read de novo assembler.** *Gigascience* 2012, **1:**18.

6. Langmead B, Salzberg SL: **Fast gapped-read alignment with Bowtie 2.** *Nature Methods* 2012, **9:**357-U354.

7. Li H, Handsaker B, Wysoker A, Fennell T, Ruan J, Homer N, Marth G, Abecasis G, Durbin R, Proc GPD: **The Sequence Alignment/Map format and SAMtools.** *Bioinformatics* 2009, **25:**2078-2079.

8. Chevreux B, Wetter T, Suhai S: **Genome Sequence Assembly Using Trace Signals and Additional Sequence Information.** *Comput Sci Biol Proc German Conf Bioinf (GCB)* 1999, **99:**45-56.

9. Zimin AV, Marcais G, Puiu D, Roberts M, Salzberg SL, Yorke JA: **The MaSuRCA genome assembler.** *Bioinformatics* 2013, **29:**2669-2677.

10. Gurevich A, Saveliev V, Vyahhi N, Tesler G: **QUAST: quality assessment tool for genome assemblies.** *Bioinformatics* 2013, **29:**1072-1075.

11. Simpson JT, Wong K, Jackman SD, Schein JE, Jones SJ, Birol I: **ABySS: a parallel assembler for short read sequence data.** *Genome Res* 2009, **19:**1117-1123.

12. Jackman SD, Vandervalk BP, Mohamadi H, Chu J, Yeo S, Hammond SA, Jahesh G, Khan H, Coombe L, Warren RL, Birol I: **ABySS 2.0: resource-efficient assembly of large genomes using a Bloom filter.** *Genome Res* 2017, **27:**768-777.

13. Hu Z, Sun C, Lu KC, Chu X, Zhao Y, Lu J, Shi J, Wei C: **EUPAN enables pan-genome studies of a large number of eukaryotic genomes.** *Bioinformatics* 2017, **33:**2408-2409.

14. Maccallum I, Przybylski D, Gnerre S, Burton J, Shlyakhter I, Gnirke A, Malek J, McKernan K, Ranade S, Shea TP, et al: **ALLPATHS 2: small genomes assembled accurately and with high continuity from short paired reads.** *Genome Biol* 2009, **10:**R103.

15. Chikhi R, Limasset A, Medvedev P: **Compacting de Bruijn graphs from sequencing data quickly and in low memory.** *Bioinformatics* 2016, **32:**i201-i208.

16. Chikhi R, Rizk G: **Space-efficient and exact de Bruijn graph representation based on a Bloom filter.** *Algorithms Mol Biol* 2013, **8:**22.

17. Kurtz S, Phillippy A, Delcher AL, Smoot M, Shumway M, Antonescu C, Salzberg SL: **Versatile and open software for comparing large genomes.** *Genome Biology* 2004, **5**.

18. Li H, Durbin R: **Fast and accurate short read alignment with Burrows-Wheeler transform.** *Bioinformatics* 2009, **25:**1754-1760.

19. Fu LM, Niu BF, Zhu ZW, Wu ST, Li WZ: **CD-HIT: accelerated for clustering the next-generation sequencing data.** *Bioinformatics* 2012, **28:**3150-3152.

20. Ye J, McGinnis S, Madden TL: **BLAST: improvements for better sequence analysis.** *Nucleic Acids Research* 2006, **34:**W6-W9.

21. Holt C, Yandell M: **MAKER2: an annotation pipeline and genome-database management tool for second-generation genome projects.** *BMC Bioinformatics* 2011, **12:**491.

22. Korf I: **Gene finding in novel genomes.** *BMC Bioinformatics* 2004, **5:**59.

23. Stanke M, Steinkamp R, Waack S, Morgenstern B: **AUGUSTUS: a web server for gene finding in eukaryotes.** *Nucleic Acids Res* 2004, **32:**W309-312.

24. Slater GS, Birney E: **Automated generation of heuristics for biological sequence comparison.** *BMC Bioinformatics* 2005, **6:**31.

25. Haas BJ, Salzberg SL, Zhu W, Pertea M, Allen JE, Orvis J, White O, Buell CR, Wortman JR: **Automated eukaryotic gene structure annotation using EVidenceModeler and the Program to Assemble Spliced Alignments.** *Genome Biol* 2008, **9:**R7.

26. Kim D, Langmead B, Salzberg SL: **HISAT: a fast spliced aligner with low memory requirements.** *Nat Methods* 2015, **12:**357-360.

27. Lan TM, Lin HX, Zhu WJ, Laurent TCAM, Yang MC, Liu X, Wang J, Wang J, Yang HM, Xu X, Guo XS: **Deep whole-genome sequencing of 90 Han Chinese genomes.** *Gigascience* 2017, **6**.

28. Mikkelsen TS, Hillier LW, Eichler EE, Zody MC, Jaffe DB, Yang SP, Enard W, Hellmann I, Lindblad-Toh K, Altheide TK, et al: **Initial sequence of the chimpanzee genome and comparison with the human genome.** *Nature* 2005, **437:**69-87.

29. Scally A, Dutheil JY, Hillier LW, Jordan GE, Goodhead I, Herrero J, Hobolth A, Lappalainen T, Mailund T, Marques-Bonet T, et al: **Insights into hominid evolution from the gorilla genome sequence.** *Nature* 2012, **483:**169-175.

30. Prufer K, Munch K, Hellmann I, Akagi K, Miller JR, Walenz B, Koren S, Sutton G, Kodira C, Winer R, et al: **The bonobo genome compared with the chimpanzee and human genomes.** *Nature* 2012, **486:**527-531.

31. Locke DP, Hillier LW, Warren WC, Worley KC, Nazareth LV, Muzny DM, Yang SP, Wang ZY, Chinwalla AT, Minx P, et al: **Comparative and demographic analysis of orang-utan genomes.** *Nature* 2011, **469:**529-533.

32. Zimin AV, Cornish AS, Maudhoo MD, Gibbs RM, Zhang X, Pandey S, Meehan DT, Wipfler K, Bosinger SE, Johnson ZP, et al: **A new rhesus macaque assembly and annotation for next-generation sequencing analyses.** *Biology Direct* 2014, **9**.

33. Rogers J, Raveendran M, Harris RA, Mailund T, Leppala K, Athanasiadis G, Schierup MH, Cheng J, Munch K, Walker JA, et al: **The comparative genomics and complex population history of Papio baboons.** *Science Advances* 2019, **5**.

34. Mallick S, Li H, Lipson M, Mathieson I, Gymrek M, Racimo F, Zhao MY, Chennagiri N, Nordenfelt S, Tandon A, et al: **The Simons Genome Diversity Project: 300 genomes from 142 diverse populations.** *Nature* 2016, **538:**201-+.
